# A Three Component Synthetic Vaccine Containing a β-Mannan T-Cell Peptide Epitope and a β-Glucan Dendritic Cell Ligand

**DOI:** 10.3390/molecules23081961

**Published:** 2018-08-06

**Authors:** David R. Bundle, Eugenia Paszkiewicz, Hassan R. H. Elsaidi, Satadru Sekhar Mandal, Susmita Sarkar

**Affiliations:** 1 Department of Chemistry, University of Alberta, Edmonton Alberta, AB T6G 2G2, Canada; eugenia.paszkiewicz@ualberta.ca (E.P.); elsaidi@ualberta.ca (H.R.H.E.); satadrusekhar@gmail.com (S.S.M.); Susmita@ualberta.ca (S.S.); 2 Department of Pharmaceutical Chemistry, Faculty of Pharmacy, University of Alexandria, El Sultan Hussein St. Azarita, Alexandria 21521, Egypt

**Keywords:** synthetic glycoconjugate vaccine, glycopeptide B and T cell epitope, β-glucan dendritic cell epitope, dectin-1 targeting, asymmetric dimeric dendrimer antigen, antibody response

## Abstract

Glycoconjugates prepared from the capsular polysaccharide of several pathogenic bacteria and carrier proteins, such as CRM 197 or tetanus toxoid, have been one of the most successful public health measures to be implemented in the last quarter century. A crucial element in the success of conjugate vaccines has been the recruitment of T-cell help and systematic induction of a secondary immune response. The seminal discovery, that degraded polysaccharide fragments with attached peptide are presented to the T-cell receptor of carbohydrate specific T-cells by MHC-II molecules that bind to the peptide component of degraded vaccine, suggests potentially novel designs for conjugate vaccines. A fully synthetic conjugate vaccine was constructed from a 1,2-linked β-mannose trisaccharide conjugated to a T-cell peptide, previously shown to afford protection against *Candida albicans*. This combined B- and T-cell epitope was synthesized with a C-terminal azidolysine residue for subsequent conjugation by click chemistry. Four copies of a β-1,3 linked hexaglucan dendritic cell epitope were conjugated to an asymmetric dendrimer bearing an alkyne terminated tether. Click chemistry of these two components created a conjugate vaccine that induced antibodies to all three epitopes of the fully synthetic construct.

## 1. Introduction

Glycoconjugates prepared from the capsular polysaccharide of several pathogenic bacteria and carrier proteins, such as CRM 197, a non-toxic mutant of diphtheria toxin or tetanus toxoid have been one of the most successful public health measures to be implemented in the last quarter century [1]. Vaccines directed against *Haemophilus influenzae*, *Streptococcus pneumoniae*, and *Neisseria meningitides* afford protection to two at-risk groups, infants and seniors, whose immune systems have not functioned well against the preceding generation of unconjugated polysaccharide vaccines [2,3,4,5]. A crucial element in the success of conjugate vaccines has been the recruitment of T-cell help and systematic induction of a secondary immune response [6,7]. Until recently, the processing and presentation of such conjugate vaccines was not well understood. The ground-breaking discovery, that degraded polysaccharide fragments with attached peptide are presented to the T-cell receptor of carbohydrate specific T-cells by MHC-II molecules that bind to the peptide component of the degraded vaccine, allowed a superior design of conjugate vaccines [6]. Instead of conjugating polysaccharide to a large carrier protein, T-cell peptide was attached to capsular polysaccharide, thereby creating a vaccine that was 50–100 times more effective in mice than a group B streptococcal glycoconjugate constructed by the technology currently used in their industrial manufacture for clinical trials.

Dendritic cells (DCs) play a crucial role in antigen uptake and processing, and targeting antigens for DC uptake has been demonstrated to hold great potential for coupling innate to adaptive immune responses [8,9,10]. Numerous applications have reported successful targeting of antigen to antigen processing cells [11,12,13], and a significant body of work has documented the use of carbohydrate ligands to mediate antigen uptake by DCs [14,15,16,17]. A variety of receptors can be employed to facilitate antigen uptake and various strategies have been proposed to enhance the affinity of carbohydrate interactions with DC receptor [18,19,20].

We have previously shown that a β-mannan trisaccharide, attached to a T-cell peptide present in a *Candida albicans* cell wall protein, afforded protection when delivered in an ex-vivo antigen-pulsed dendritic cell-based vaccine strategy [21]. The same glycopeptide, when conjugated to tetanus toxoid, afforded protection without the use of specialized immunization protocols or adjuvants [22]. Targeting this type of conjugate vaccine to the dectin-1 receptor of dendritic cells by covalent attachment of a β-glucan to the glycopeptide-protein conjugate resulted in an enhanced antibody response to the β-mannan epitope of the conjugate [23].

Extending these findings and inspired by the work on glycopeptide presentation to T-cells [6], we envisioned a fully synthetic β-mannan glycopeptide conjugate that would utilize targeting of antigen to dendritic cells as a way to offset the anticipated diminished T-cell response that was likely to result from the absence of multiple T-cell peptides generated from a large protein carrier such as tetanus toxoid used in our previous work. Based on our earlier work, we reasoned that DC targeting of a fully synthetic carbohydrate-peptide vaccine would compensate for otherwise low immunogenicity.

**Figure 1:**
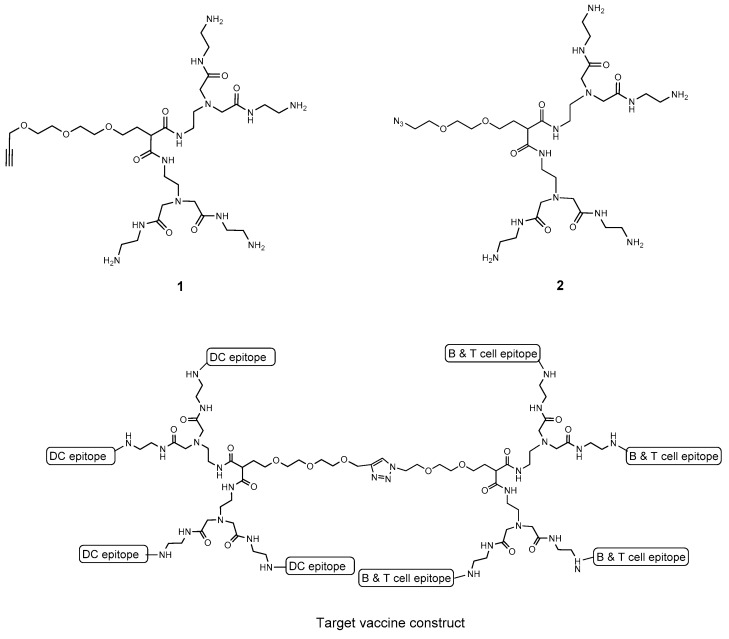
Two asymmetric dendrimers for display of dendritic cell targeting β-glucan on **1** and presentation of combined B- and T-cell epitopes on **2**.

**Figure 2:**
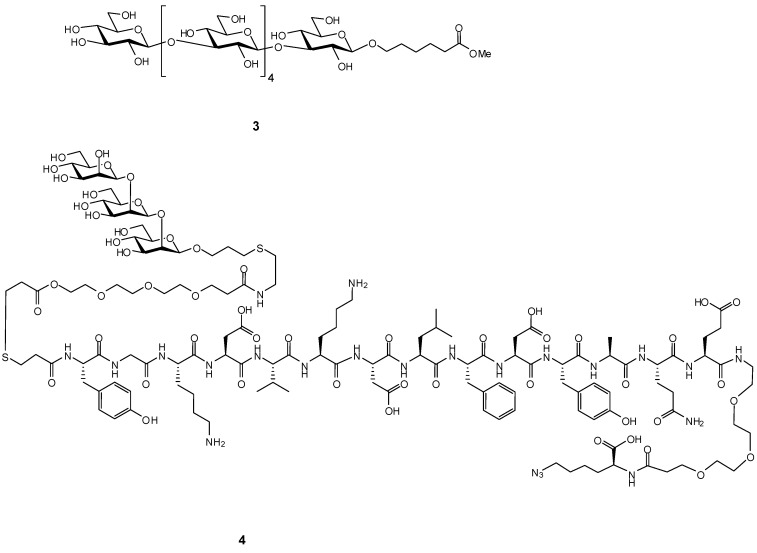
The dendritic cell epitope **3** which is taken up by dectin-1 and the combined B- and T-cell epitope **4** consisting of the *C. albicans* cell wall β1,2-mannotriose and the cell wall peptide Fba.

Since glycan receptor binding affinities are intrinsically weak when compared to those between peptides and proteins, we chose to design asymmetric dendrimers **1** and **2** (Figure 1), to which B- and T-cell epitopes and dendritic cell epitopes could be attached in multiple copies, to exploit avidity gains to enhance receptor uptake. We elected a modular design which employed two asymmetric dendrimers, one carrying a β1,3 hexaglucan ligand **3** [24] (Figure 2) specific for the dectin-1 dendritic cell receptor. This choice was based on literature precedence. Several studies have investigated the interaction between glucan oligosaccharides and dectin-1 [18,19,25,26]. The exact size of the glucan required for binding has been the subject of differing conclusions. However, NMR studies show the minimal size epitope corresponds to a hexasaccharide [25,26] and multimeric presentation of a hexasaccharide has been reported to be highly effective for vaccine uptake [20]. The second dendrimer would carry the covalently attached B- and T-cell epitopes **4** (Figure 2). Click chemistry would be used to fuse the two halves to create a totally synthetic vaccine construct. A variety of conjugation chemistries were considered for attachment of haptens and ligands to the arms of the two dendrimers with the requirement of high conjugation efficiency, ideally for stoichiometric amounts of dendrimer and hapten/ligand.

We report our attempts to reduce these ideas to practice using the *C. albicans* B-cell β1,2-mannotriose epitope attached to the Fba peptide T-cell epitope, glycopeptide **4** presented on dendrimer **2**, a dectin-1 ligand, β1,3 glucan hexasaccharide **3**, displayed on dendrimer **1**, and the corresponding immune response of mice to a fully synthetic conjugate prepared from these components.

## 2. Results

### 2.1. Synthesis of Dendrimers

We elected to synthesize two asymmetric dendrimers with four carboxylic amide terminated arms and a fifth polyethylene glycol arm terminated by an azide or alkyne. After decoration of the amide derivatives with the various epitopes, the two dendrimer halves could be conjugated to provide a molecule equipped with the three essential elements B-cell, T-cell, and dendritic cell epitopes. If successful, this modular design would allow selection of optimum pairing of these individual elements. Alternatively, the asymmetric dendrimers might be conjugated individually to carrier protein and provide four ligands per substitution site, thereby minimizing masking of T-cell peptide epitopes that would occur if high loading of haptens is desired.

**Scheme 1:**
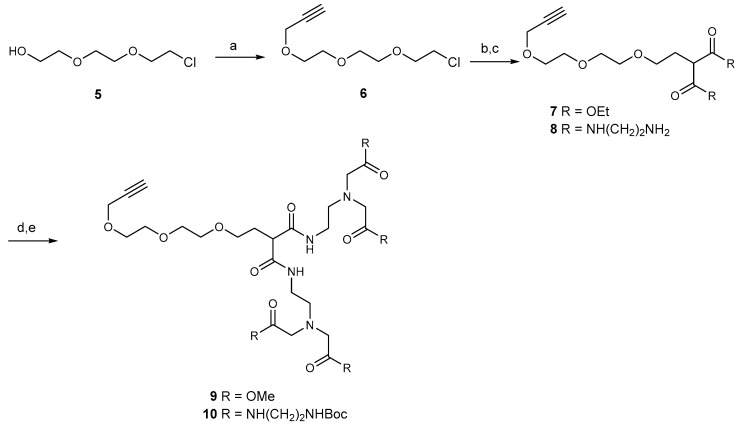
(a) CHCCH_2_Br, NaH, DMF; (b) CH_2_(CO_2_Et)_2_, NaH, THF; (c) NH_2_CH_2_CH_2_NH_2_, 50 °C for 48 h; (d) MeCN, Na_2_CO_3_, BrCH_2_CO_2_Me, 60 °C for 2 h; (e) (i) NH_2_CH_2_CH_2_NH_2_, 50 °C for 3 d. (ii) Boc_2_O, MeOH/Et_3_N.

Both dendrimers were synthesized by Michael addition of malonate to suitably protected triethylene glycol derivatives. The propargyl group was introduced to the chloro derivative **5** to provide **6**. Displacement of chloride ion by the anion of diethyl malonate provided **7**, which was converted to amide **8** (Scheme 1). Alkylation of **8** with methyl bromoacetate gave the ester **9**, and it was converted to crude **1** by reaction with ethylene diamine. However, significant traces of diamine could not be removed from the product, and the mixture of crude **1** and amine was converted to the corresponding BOC derivative **10** and the BOC derivative of ethylene diamine. This allowed chromatographic purification of **10**. Treatment of **10** with TFA afforded pure dendrimer **1**.

**Scheme 2:**
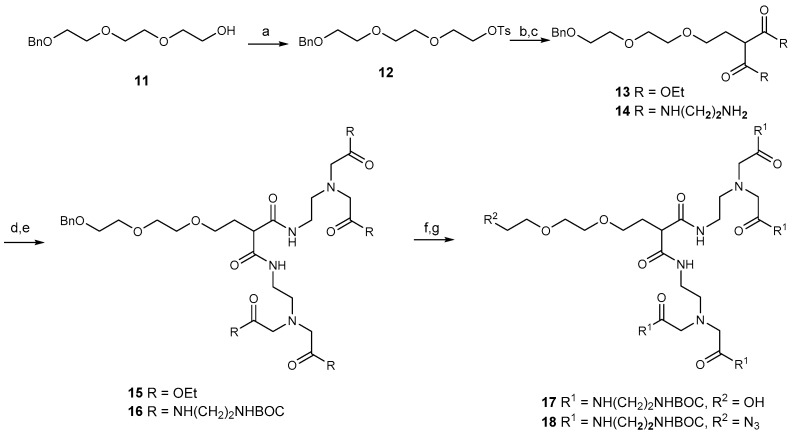
(a) TsCl, DCM/Et_3_N; (b) CH_2_(CO_2_Et)_2_, NaH, DMF; (c) NH_2_CH_2_CH_2_NH_2_, 50 °C for 48 h; (d) MeCN, Na_2_CO_3_, BrCH_2_CO_2_Me, 60 °C for 18 h; (e): (i) NH_2_CH_2_CH_2_NH_2_, 50 °C for 48 h, (ii) Boc_2_O, DCM/Et_3_N; (f) H_2_/Pd(OH)_2_/DCM 21 °C for 16 h; (g):(i) MsCl, DCM, Et_3_N, (ii) NaN_3_, DMF 55 °C for 2 h.

Preparation of dendrimer **2** used benzyl ether **11**, prepared according to a published procedure [27] and then converted to the tosyl ester **12**. Displacement of the tosyl group by malonate anion gave **13**, which was converted to amide **14**. Reaction with ethyl bromoacetate gave **15** and this was converted to an amide with ethylene diamine and without isolating the amide, the amine groups were protected as their BOC derivative **16**. Hydrogenation of **16** gave the alcohol **17**. Mesylation gave a 90% yield of the mono-mesylate, and reaction with sodium azide in DMF afforded azide **18** in 80% yield (Scheme 2). Removal of the BOC groups under standard conditions gave pure dendrimer **2**.

### 2.2. Synthesis of the Tetravalent Dectin-1 Ligand

**Scheme 3:**
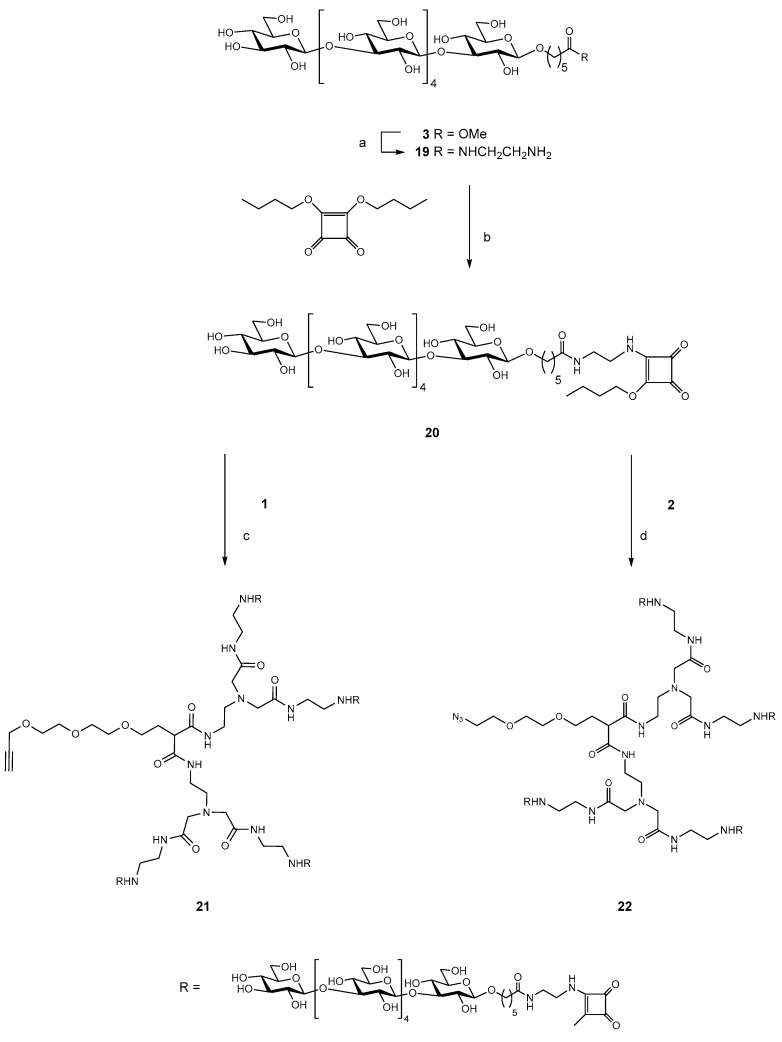
(a) NH_2_CH_2_CH_2_NH_2_, 50 °C for 18 h; (b) EtOH/H_2_O NaHCO_3_, pH 8 30 min.; (c) 0.5 M borate buffer, pH 9, 18 °C 3 d.; (d) 0.5 M borate buffer, pH 9, 18 °C 2 d.

The hexasaccharide methyl ester **3** [24] was converted to the amide **19** and then activated by reaction with dibutyl squarate [28,29] to give the half ester **20**, which was purified by HPLC. Six molar equivalents of **20** were reacted with 1 mole equivalent of the alkyne dendrimer **1** to give the fully substituted target dendrimer **21** in 78% yield. An approximately six-fold molar excess of the same activated hexasaccharide half ester **20** was conjugated to azide dendrimer **2** to give the fully substituted azide dendrimer **22** in 72% yield (Scheme 3).

### 2.3. Synthesis of B- and T-Cell Epitope, β-Mannotriose Fba Glycopeptide

**Scheme 4:**
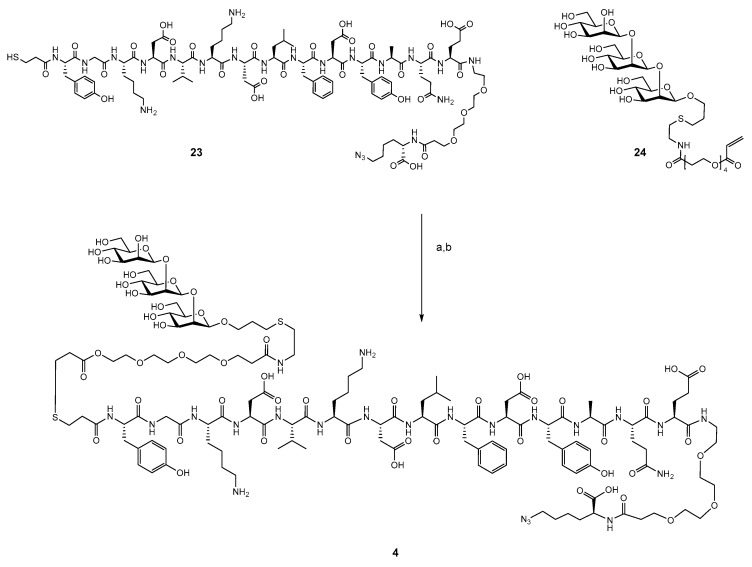
(a) MeCN/H_2_O, 0.02 M borate buffer pH 8.15, 5 h. (b) HOAc, HPLC.

The previously reported approach for synthesis of the β-mannotriose Fba glycopeptide was employed here with the modification of a C-terminal azido lysine residue [30,31]. This peptide was synthesized by standard solid phase chemistry. After addition of the final tyrosine residue, the peptide was capped by *S*-tritylthiopropionic acid. The completed peptide **23** was cleaved from the resin and purified by reverse phase HPLC. β-Man_3_-acrylate **24** was conjugated to peptide **23** by a previously reported method (Scheme 4) to provide glycopeptide **4** in 56% yield [30] (Supplementary Material, Figure S1).

### 2.4. Attempts to Conjugate Glycopeptide 4 to Dendrimer

Our initial approach was to conjugate excess β-mannotriose Fba glycopeptide (Man_3_-Fba) to the propargyl dendrimer **1** to provide a tetrameric dendrimer of the glycopeptide (schematic structure, Figure 1). We anticipated that to achieve close to stoichiometric coupling of the dendrimer and glycopeptide, a reactive pairing would be optimal. For this purpose, we chose a bromoacetate reacting with the thiol. We intended to use a form of the Man_3_-Fba glycopeptideas a C-terminal thiol (C-terminal cysteine) which we have described in previous work [30,31]. Activation of **1** as a tetrabromoacetate was attempted, but this proved to be challenging. Reactions of **1** with bromoacetyl NHS ester appeared to proceed well, but on closer examination by MALDI MS, the initially formed dendrimer bromoacetate reacted with *N*-hydroxysuccinimide. We then used bromoacetyl bromide to activate **1**. This worked well, but since the glycopeptide was protected as an *S*-acetate, it was necessary to use mild base to remove acetate. When we used hydroxylamine for this purpose, we obtained a trisubstituted glycopeptide dendrimer with the fourth arm substituted by hydroxylamine.

To avoid reaction of bromoacetate with hydroxylamine peptide, *S*-acetyl was first cleaved, and the thiol was purified prior to reaction with dendrimer bromoacetate. The crude reaction product contained a significant amount of peptide disulfide and many other impurities, in addition to only minor quantities of a trisubstituted and tetrasubstituted dendrimer. It became clear that this approach could not provide the target material in acceptable yield, and we opted to directly conjugate the glycopeptide to dendrimer **1**.

### 2.5. Conjugation of Glycopeptide 4 with the Tetravalent β-Glucan Dendrimer 21

**Scheme 5:**
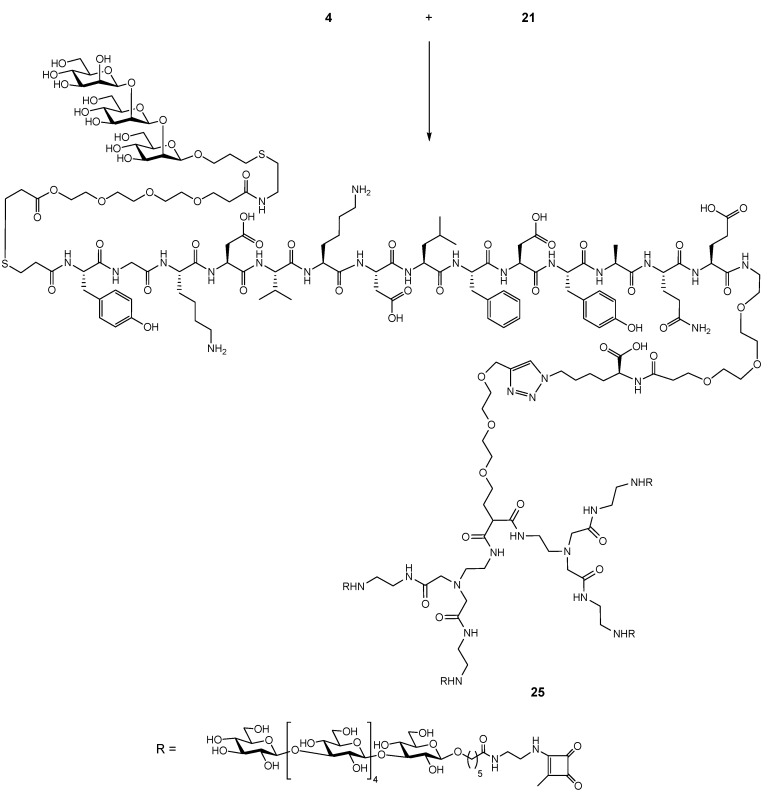
(a):(i) H_2_O, Cu, 0.2 M Tris buffer pH 8, bathophenantroline Cu^+1^ catalyst [31,34], (ii) 0.5 M EDTA pH 8, HPLC GlycanPac AXH-1.

Attempts to conjugate hexasaccharide dendrimer **21** and glycopeptide **4** using standard click reaction conditions with copper sulfate and sodium ascorbate [32,33] did not give the expected result. LC-MS analysis of the reaction mixture suggested hydrolysis of propargyl ether (RCH_2_–O–CH_2_–CCH → RCH_2_–OH), since the molecular weight of the dendrimer component was 38 Da lower than that of **21**. Conjugation was successful when Cu^+1^ bathophenantroline catalyst [34,35] was used in Tris buffer pH 8 under oxygen free conditions, giving the final product **25** (Scheme 5).

The crude product required several purification steps. First, after EDTA treatment, the reaction mixture was spun to remove solids, then the solution was dialyzed against degassed deionized water using an Amicon centrifugal filter (3000 MWCO), and finally, after lyophilization, it was purified on a GlycanPack-AXH-1 analytical column using 0.1 M aqueous ammonium buffer and acetonitrile–water as eluents, with multiple injections and collection of small fractions (0.5 mL) which were analyzed by MALDI TOF MS. Fractions containing product with the required molecular weight were pooled and lyophilized several times to remove volatile ammonium formate. The conjugate **25** was analyzed by LC/UV/ESI-MS (Supplementary Material, Figure S2).

### 2.6. Conjugation of β-Glucan Dendrimer 22 to Activated Ovalbumin

A solution of ovalbumin was activated by *N*-acylation with 3-prop-2-ynyloxy-propionic acid 2,5-dioxo-pyrrolidin-1-yl ester. The product bearing an average of two propargyl groups was conjugated with dendrimer **22** by click chemistry [34,35] (Supplementary Material, Scheme S1). The resulting conjugate contained an average of one dendrimer per ovalbumin. This product was used for ELISA to detect β-glucan specific antibodies.

### 2.7. Immunization of Mice with the Trivalent Antigen 25 and Assay of Antibody Profiles

Mice were immunized with dendrimer glycopeptide conjugate **25** three times at 21-day intervals. The antigen was prepared in PBS and mixed with an equal volume of Freund’s incomplete adjuvant. Each mouse received 12.5 μg/injection which corresponds to approximately 0.3 μg of mannotriose and 1.2 μg of Fba peptide.

For the purposes of comparison of the immune response to **25** with the response to a glycopeptide conjugated to tetanus toxoid, a conventional carrier protein, we immunized mice with the same glycopeptide conjugated to tetanus toxoid (TT), prepared as previously described [21]. Five mice were immunized with this antigen given with alum, and another five were immunized without alum. The mice in these two experiments also received a dose equivalent to 0.3 μg of mannotriose and 1.2 μg of Fba peptide.

Trial bleeds were taken 10 days after the first immunization, and a final bleed was collected 10 days after the third immunization. Sera from first and final bleeds were screened against four conjugates, mannotriose conjugated to bovine serum albumin (BSA) (Man_3_-BSA) [23], peptide Fba conjugated to polyvinylpyrolidone (Fba–PVP) [36,37], β-glucan hexasaccharide attached to dendrimer and conjugated to ovalbumin (β-Glc–Dend–OVA) and β-glucan hexasaccharide conjugated to BSA (Hexa–BSA) via the disuccinimidyl glutarate (DSG) linker. ELISA titration data is provided in Supplementary Material (Figures S3–S10).

**Figure 3:**
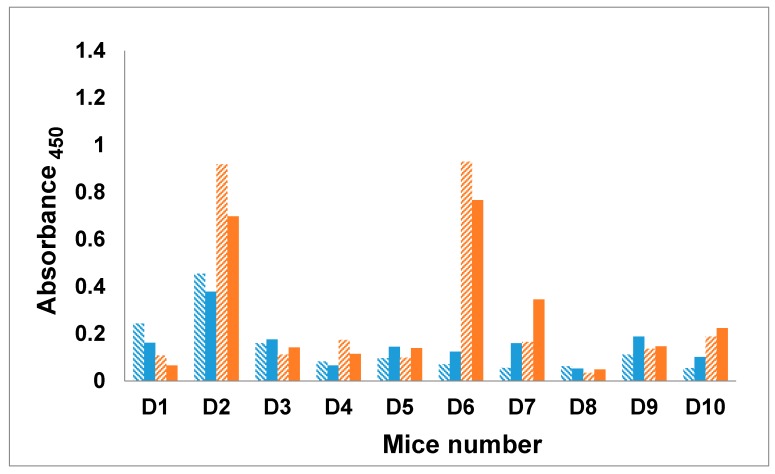
Antibody profile of murine sera vaccinated with dendrimer–glycopeptide conjugate titrated against Man_3_–BSA antigen. All sera were diluted 1:1000. Blue colored bars denote IgM response and orange colored bars the IgG response. Solid bars represent final bleed, whereas hashed bars denote first bleed. All the data points were plotted after subtracting the background value consisting of all assay reagents but sera.

The IgM and IgG antibody titers measured against Man_3_–BSA showed a good response in two mice (D2 and D6), but an otherwise weak to negligible response in the remaining eight mice (Figure 3). Four mice (D2, D6, D7, and D10) developed a secondary immune response with an IgM to IgG class switch, however, the two highest responders (D2 and D6) showed a small drop in IgG level at final bleed. The remaining mice exhibited comparable IgM and IgG titers, and in one case, higher IgM response. In all of these examples, at a dilution of 1:1000, the antibody titers were at or close to their endpoint (Figures S3–S10, Supplementary Material).

**Figure 4:**
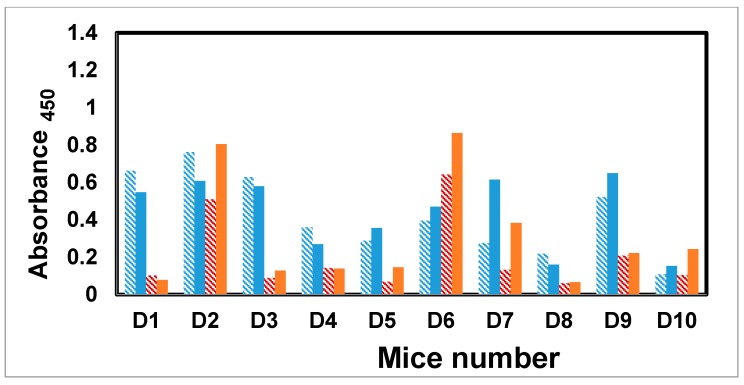
Antibody profile of murine sera vaccinated with dendrimer–glycopeptide conjugate when titrated against the Fba–PVP. All the sera were diluted 1:1000. The blue colored bars denote IgM response, whereas red colored bars represent IgG response. Under each category, solid bars represent final bleed whereas hashed bars denote first bleed. All the data points were plotted after subtracting the background value consisting of all assay reagents but sera.

A moderate IgM and IgG antibody response was detected against the Fba peptide after the first immunization in all but two mice (D8 and D10). As with the response to the mannotriose epitope, mice D2 and D6 also showed an IgM to IgG class switch and elevated secondary response (Figure 4). For the remaining mice that responded above background (D1, D3, D4, D5, D7, and D9), IgM titers were greater than IgG.

**Figure 5:**
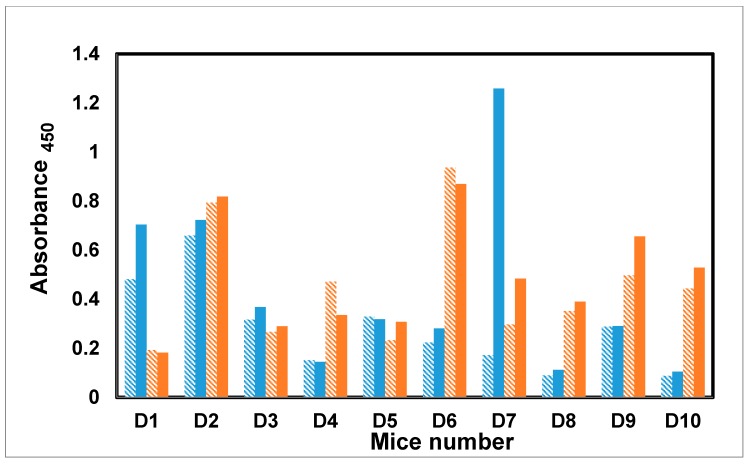
Antibody profile of murine sera vaccinated with dendrimer–glycopeptide conjugate **25** when titrated against the dendrimer–hexasaccharide (all the sera were diluted 1:1000). The blue colored bars denote IgM response whereas red colored bars represent IgG response. Under each category, solid bars represent final bleed, whereas hashed bars denote first bleed. All the data points were plotted after subtracting the background value consisting of all assay reagents but sera.

A good antibody response was detected against the dendrimer hexasaccharide (Hexa–Dendrimer–OVA). Six mice, with the exception of D1, D3, D5, and D7, exhibited a class switch with elevated IgG titers (Figure 5).

**Figure 6:**
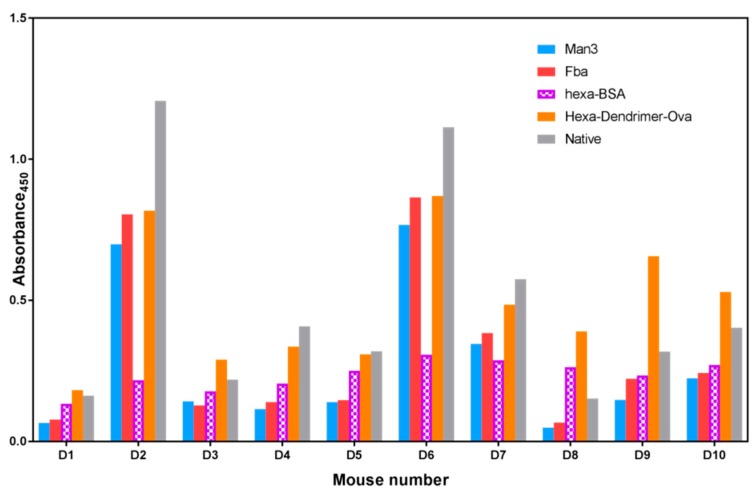
IgG profile of the final bleed collected from mice vaccinated with dendrimer–glycopeptide conjugate when titrated against various antigens. All the sera were diluted 1:1000. The blue colored bars denote mannotriose response, the red bar denotes Fba response, the blue bar the response to synthetic β-glucan, the orange bar denotes dendrimer–hexasaccharide response, while the grey bar denotes native antigen response. All the data points were plotted after subtracting the background value consisting of all assay reagents but sera.

The IgG titers measured for the final bleed of all ten mice are compared for each of the three epitopes and the native *C. albicans* antigen, a cell wall extract enriched for carbohydrate antigens (Figure 6) [38] In order to distinguish a squarate response from a β-glucan response, a fourth antigen was screened. Hexa–BSA is composed of hexasaccharide conjugated to BSA via the DSG linker. In all ten mice, the response to the Hexa–Dendrimer–OVA bearing four to eight copies of hexasaccharide per OVA are higher than the response to Hexa–BSA, which has a similar hexasaccharide loading. This suggests the presence of an anti-squarate response, with the caveat that clustered dendrimer and random BSA displayed epitopes are equally antigenic in ELISA. Titers against the *C. albicans* cell wall extract, which contains β-mannan and β-glucan epitopes but not Fba, exhibited the highest antibody response in mice D2, D4, D5, D6, and D7. In the other five mice, the response measured against the β-glucan–dendrimer–ovalbumin had the highest titers.

**Figure 7:**
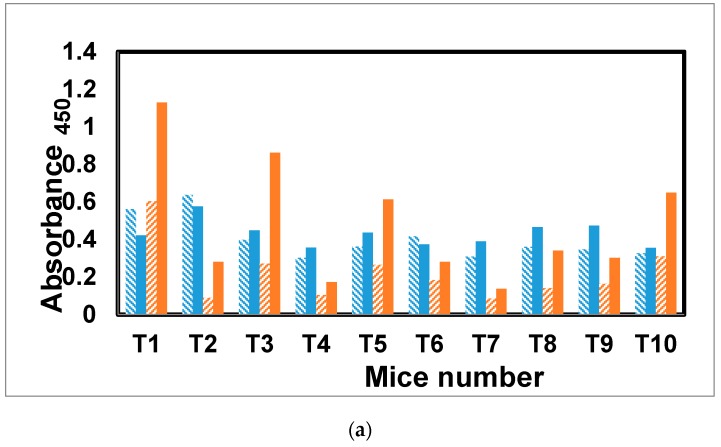

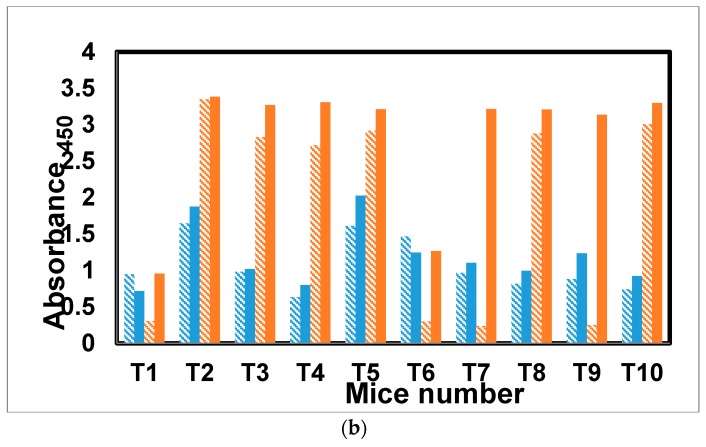
(**a**) Antibody profile of murine sera vaccinated with tetanus toxoid–glycopeptide conjugate when titrated against the Man_3_–BSA. All the sera were diluted 1:1000. The blue colored bars denote IgM response, whereas red colored bars represent IgG response. Under each category, solid bars represent final bleeds, whereas hashed bars denote first bleed. All the data points were plotted after subtracting the background value from similarly diluted pre-bleed sera. (**b**) Antibody profile of murine sera vaccinated with tetanus toxoid–glycopeptide conjugate when titrated against PVP–Fba. All the sera were diluted 1:1000. The blue colored bars denote IgM response, whereas red colored bars represent IgG response. Under each category, solid bars represent final bleed, whereas hashed bars denote first bleed. All the data points were plotted after subtracting the background value consisting of all assay reagents but sera.

The antibody response to glycopeptide tetanus toxoid conjugate [31] was evaluated in a similar fashion to mice receiving the dendrimer antigen **25**. Of the five mice receiving the antigen with alum, three showed a class switch (T1, T3, and T5) and elevated secondary response for antibodies specific for mannotriose (Figure 7a,b). Only one mouse, T10 from the group receiving antigen without alum, showed a class switch and elevated secondary response. The other four mice, T6–T9, as well as T2 and T4 from the first group, all had lower IgG than IgM antibody levels in the final versus first bleeds.

In this conjugate, the response to the Fba peptide stands in sharp contrast to the β-mannan response. Only one mouse from each group (T1 and T6) failed to show strong antibody titers and a pronounced IgM to IgG class switch for the peptide.

**Figure 8:**
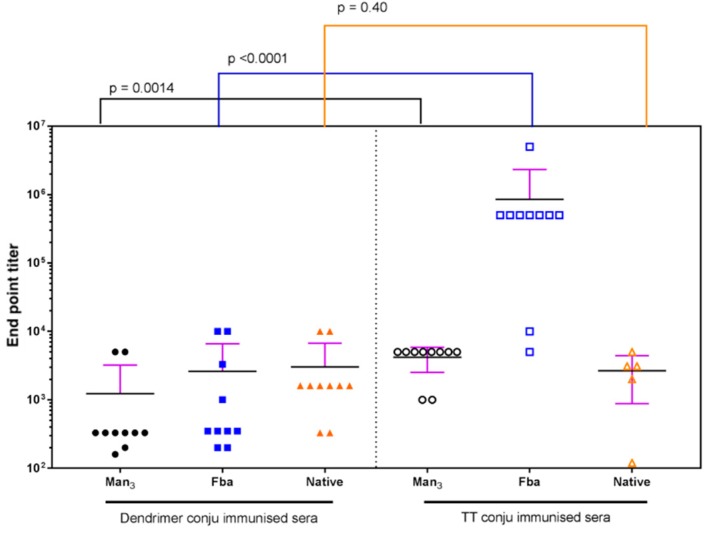
Comparison of the antibody response measured as endpoint titers to dendrimer conjugate **25** with the response to the glycopeptide tetanus toxoid conjugate measured against the three antigens, Man_3_–BSA, Fba–PVP, and native antigen extract. Calculated *p* values indicate that the tetanus toxoid conjugate induced higher antibody titers to Man_3_ and Fba epitopes.

Finally, the antibody response to **25** and the tetanus toxoid conjugate are compared for the final bleed endpoint titers against three epitopes Man_3_–BSA, Fba–PVP, and the *C. albicans* cell wall extract (Figure 8). The glycopeptide tetanus toxoid conjugate induces statistically high titers against Man_3_ and significantly higher Fba antibody levels than the dendrimer immunogen **25**. Titers against the *C. albicans* cell wall extract are not statistically different. While mice immunized with **25** showed a clear response to Man_3_ and β-glucan hexasaccharide (Figure 3, Figure 5 and Figure 6) mice immunized with the glycopeptide tetanus toxoid conjugate possessed high pre-immune titers against the native antigen. This precludes a comparison of the response to cell wall extract.

## 3. Discussion

Our attempts to create a fully synthetic antigen by conjugating two asymmetric dendrimers bearing alternatively four DC β-glucan epitopes and four B and T cell glycopeptide epitopes could not be completed in the configuration originally envisaged. Conjugation of four glycopeptides to dendrimer **1**, by chemistry that seemed likely to avoid creation of neoepitopes, was not sufficiently robust to provide good yields of fully substituted dendrimer. Separation of di, tri, and tetrasubstituted dendrimers becomes a major hurdle to be overcome, and underlines the imperativeness for quantitative substitution of the dendrimer. In our hands, when we created an activated dendrimer with bromoacetate groups, these were highly reactive and difficult to handle. The corresponding thiol group of the glycopeptide proved to be too prone to disulfide bond formation, and attempts to reduce this to generate thiol during conjugation did not result in a conjugated product in acceptable yield.

Squarate chemistry proved highly effective in preparation of dendrimer **2** bearing four copies of the β-glucan hexasaccharide, resulting in conjugate **21**. Conjugation of low molecular weight epitopes via the squarate functional group seems more likely to create neoepitopes, even though this is not observed with larger polysaccharide antigens [39,40].

We elected to conjugate glycopeptide **4** to **21** resulting in the conjugate **25**. However, this creates a numerical imbalance; a fourfold higher abundance of β-glucan DC epitope over the mannotriose and Fba peptide B- and T-cell epitopes. The immune response against Man_3_ and β-glucan in mice immunized with **25** reflects this effective concentration difference.

The availability of conjugates displaying individual epitopes allowed the antibody response to conjugate **25** and the glycopeptide tetanus toxoid conjugate to be dissected. The response to Man_3_ was weak in both conjugates, and only slightly higher in the glycopeptide tetanus toxoid-immunized mice (Figure 8). The response to the Fba peptide was higher for both antigens, and notably higher in the tetanus toxoid conjugate, suggesting this epitope is more immunogenic.

Antibody levels are higher against the β-glucan epitope, with its fourfold higher concentration in the immunizing antigen **25**, than those against mannotriose. With regard to potential neoepitopes created by squarate groups titers against Hex–BSA are lower than those measured against β-Glc–Dend–OVA. This is consistent with antibodies specific for neoepitopes present in the β-glucan–squarate–dendrimer segment of **25**.

While the response to immunization of 10 mice with dendrimer conjugate **25** was generally poor, when comparing the antibody response of the three highest responding mice (D2, D6, and D7) with results from our previous studies [21,22], the fully synthetic antigen **25** generates antibody titers to Man_3_ similar to those achieved in live challenge experiments [20,21]. Despite the flaws in the synthetic design of **25**, which includes a larger number of DC targeting epitopes, this antigen was moderately successful as a *C. albicans* specific immunogen. As noted above, β-glucans are important protective fungal antigens [41,42], so our design of **25** had the fortuitous effect of generating additional potentially protective antibodies.

We have not investigated the cellular immune response in this study as we did in other work [23], and cannot know whether the DC targeting by the β-glucan hexasaccharide epitope enhanced the response to **25**. When we targeted a mannotriose tetanus conjugate for DC uptake by conjugating laminarin to the glycoconjugate, we observed a 10-fold improvement in antibody levels to the β-mannan, and established clear evidence for activation of DCs [23]. Literature reports suggest that the β-glucan hexasaccharide epitope are effective in promoting DC uptake [20], and vaccines based on β-glucans have been reported as providing protection against *C. albicans* [41,42].

## 4. Materials and Methods

Details of chemical synthesis and the characterization of products are supplied in Supplementary Material.

### 4.1. Animals

Female Balb/c mice (Charles River, Canada) of 6–8 weeks old were used to study the immune response (ethic approval number: AUP00000090). All the procedures and experiments involving animals were carried out using a protocol approved by the Animal Care Committee, Faculty of Bioscience, University of Alberta. The protocol was approved as per the Canadian Council on Animal Care (CCAC) guidelines.

### 4.2. Antigens

The dendrimer conjugate **25** and a previously reported tetanus toxoid glycopeptide conjugate [31] were used as immunizing antigens. Three different antigens, Man_3_–BSA, Fba–PVP conjugate, and dendrimer–hexasaccharide–ovalbumin conjugate, described here, were used for ELISA assay. An additional conjugate, Hexa–BSA, was prepared by conjugating the hexasaccharide xx to BSA via the homo bifunctional linker disuccinimidyl glutarate, DSG, (unpublished results). Sera were also screened against the native antigen, extracted from the cell wall of *Candida albican* (a kind gift from Dr J. E. Cutler) [21].

### 4.3. Vaccine Formulation

A solution of dendrimer 25 (100 μg/mL in PBS) was emulsified with an equal volume of Freund’s incomplete adjuvant. Each mouse received 12.5 microgram/injection (which corresponds to approximately 0.3 μg of mannotriose and 1.2 μg of Fba. Tetanus toxoid conjugate was administered with and without alum. Toxoid conjugate (100 μg /mL in PBS) was mixed with freshly prepared alum (50 μL of 500 μg /mL suspension) and 150 mL of this suspension was administered to each mouse. The PBS solution toxoid conjugate was diluted to 40 μg /mL and 250 μL of this solution was used to immunize mice. Five of the animals were immunized with alum, and the other five without alum. All the mice received a dose equivalent to 0.3 μg of mannotriose and 1.2 μg of Fba.

### 4.4. Immunization

Balb/c mice were immunized three times at a 21-day interval. A total volume of 250 µL was injected to each mouse—150 µL interperitoneally, and 100 µL was injected subcutaneously. Pre-bleeds were collected before the immunization started, and the first test bleeds were collected 10 days after the first immunization. Mice were euthanized 10 days after the final injection, and final bleeds were collected.

### 4.5. Serum Processing

After collection, murine blood was incubated at 37° C for one hour, then spun at 1500 *g* for 10 min. Clear serum form the top was collected and stored at −20 °C until use.

### 4.6. Immunoassays

Antibody levels in murine sera were studied using enzyme linked immunosorbent assay (ELISA). A published protocol was followed with little modification [37]. Briefly, polystyrene microtiter plates were incubated with the coating antigen (1 μg/mL, 100 μL/well) at 4 °C overnight, then washed (5×) with PBST (0.05% Tween-20 in phosphate buffer saline, PBS). Then, murine sera diluted 1000-fold were added to the coated wells (100 μL/well). After incubation at room temperature for 2 h, the plates were washed (5×) with PBST. Then, the plate was incubated with 100 μL/well of 1:5000 horse radish peroxidase labelled goat anti-mouse IgG antibody (KPL, 1.0 mg/mL stock) for 30 min at room temperature, then washed (5×) with PBST. A peroxidase substrate, 3,3′,5,5′-tetramethylbenzidine (TMB) with H_2_O_2_, was added. After 15 min, the reaction was quenched by addition of phosphoric acid (1 M, 100 μL/well). The plates were read at 450 nm, and the data were processed using Origin software (Origin 2017, OriginLab Corporation, Northampton, MA, USA). The buffer, 0.1% BSA in PBST was used to dilute all sera. Endpoint dilution (x_0_) was recorded as the serum dilution giving an absorbance 0.2 above background, and serum titers was calculated as the reciprocal of x_0_. All data were plotted using Prism software (Version 7, GraphPad Software, Inc., LaJolla, CA, USA).

## 5. Conclusions

The challenges we encountered in constructing a dendrimer-based synthetic immunogen suggest that dendrimers are not the most attractive candidate for further work on fully synthetic vaccines. The requirement to achieve full substitution on all arms of the dendrimer could not be reached for the B- and T-cell epitopes, and purification and analysis of such large molecular weight entities are not trivial. A potentially attractive approach could involve the use of suitably designed coiled-coils that incorporate amino acids for specific conjugation sites. High affinity non-covalent association of two differently substituted coils is an attractive alternative to chemical conjugation of two already large molecules. A tricomponent immunopotentiating system for malaria vaccine development using this approach has been reported [13] and self-assembly methods have great appeal and flexibility [43,44]. The success of a conjugate vaccine in which T-cell peptides are conjugated to a polysaccharide is substantially more protective in a neonatal mouse model of group B Streptococcus infection polysaccharide-protein conjugates [6]. Our results suggest that any synthetic construct should contain larger copy numbers of both B- and T-cell epitopes than the DC targeting ligand.

## Supplementary Materials

The following are available online. Detailed synthetic methods, Figures and Schemes and NMR spectra; ELISA Titration data and Figures S3–S7.
