# Supplementary material for: A Three Component Synthetic Vaccine Containing a β-Mannan T-Cell Peptide Epitope and a β-Glucan Dendritic Cell Ligand
Source: Molecules. 2018 Aug 6;23(8):1961. doi: 10.3390/molecules23081961 (PMC6222438; doi:10.3390/molecules23081961)
Supplement: Supplementary file 1 [file molecules-23-01961-s001.pdf]

David R. Bundle[1], Eugenia Paszkiewicz[1], Hassan R. H. Elsaidi[1,2], Satadru Sekhar Mandal[1,3], and Susmita Sarkar[1]

[1] Department of Chemistry, University of Alberta, Edmonton Alberta, AB T6G 2G2 Canada; eugenia.paszkiewicz@ualberta.ca (E.P.); elsaidi@ualberta.ca (H.R.H.E.); satadrusekhar@gmail.com (S.S.M.); Susmita@ualberta.ca (S.S.)
[2] Department of Pharmaceutical Chemistry, Faculty of Pharmacy, University of Alexandria, El Sultan Hussein St. Azarita, Alexandria, 21521, Egypt

* Correspondencedave.bundle@ualberta.ca; Tel.: +01-780-492-8808

† Current address: TCG Lifesciences Prt. Ltd., BN Block, Plot-7, Sector-V, Salt lake, Kolkata-700091, WB, India

Supplementary Material

Experimental

Reagents

Dichloromethane (DCM), *N*,*N*-dimethylformamide (DMF) and methanol (MeOH) were obtained from Fisher Scientific. Acetonitrile 190 (CH_3CN) for HPLC were purchased from Caledon Labs. Diethyl ether, *N*,*N*-diisopropylethylamine (DIPEA), triisopropyl silane (TIPS) and piperidine were obtained from Sigma-Aldrich. Hydroxybenzotriazole (HOBt) and O-benzotriazole-*N*,*N*,*N'*,*N'*-tetramethyluronium hexafluorophosphate (HBTU) were purchased from Matrix Innovation. *N*-Fmoc-Lys(N_3)-OH were purchased from ChemPep Inc., *N*-methylpyrrolidone (NMP), 2-chlorotrityl chloride resin, N^{α}-Fmoc-amino acids and FmocNH(CH_2CH_2O)$_3$(CH_2)$_2$COOH (Fmoc-tPeg-OH) were obtained from Advanced ChemTech., trifluoroacidic acid (TFA) from Oakwood Chemicals. Solvents were dried using a Pure-Solv purification system from Innovative Technology. Deionized water was delivered from Mili-Q water filtration system (Milipore Co.).

Purification and characterization

HPLC purifications were performed on Waters Delta 600 HPLC system equipped with Empower 2 software and Waters 2996 photodiode array detector. Analytical and preparative separations employed Phenomex Luna C18 (2) 5 μm HPLC columns: analytical (250 x 4.6 mm), flow rate 1mL/min; semi preparative (250 x 10 mm), flow rate 5 mL/min and preparative (250 x 21.5 mm), flow rate 10 mL/min applying linear gradient of solvents A and B (A: H2O, B: CH_3CN, usually with addition of AcOH or TFA). Purification of the dendrimer-glycopeptide construct was carried on GlycanPac AXH-1 (3 μm, 150 x 4.6 mm) analytical column (Dionex/ ThermoFisher Scientific). UV absorptions at 212 and 280 nm were used for eluted compounds detection. Mass spectrometry detection for compounds identification was carried on AB Sciex Voyager Elite MALDI mass spectrometer using Matrix Assisted Laser Desorption/Ionization (MALDI) – Time of Flight Mass Spectrometry (TOF MS). Samples were prepared on a stainless steel sample plate and DHB (2.5-dihydroxybenzoic acid) or sinapinic acid (4-hydroxy-3,5-dimethoxy-cinnamic acid) was used as a matrix. HPLC-UV-MS was performed using an Agilent 1200 SL HPLC System with a Kinetex 1.7µm particle size, EVO C18, 2.1x50 mm, 100Å, reverse phase analytical column with a buffer gradient system of 0.1% formic acid (FA) in water as mobile phase A and 0.1% FA in acetonitrile as mobile phase B or GlycanPac AXH-1 (1.9 μm, 2.1 x 100 mm) analytical column (Dionex/Thermo) with a buffer gradient of 80% acetonitrile and 20% water as A solvent and 0.1 M ammonium formate (AF) pH 4.4 as solvent B. UV detection was recorded at 214, 254 or 280 nm. Mass spectra were acquired in positive mode ionization using an Agilent 6220 Accurate-Mass TOF HPLC/MS system. Analysis of the HPLC-MS data was done using the Agilent Mass Hunter Qualitative Analysis software. MS/MS analysis was carried on a Waters (Micromass) Q-TOF Premier mass spectrometer. ^{1}H NMR spectra were recorded at 600 or 700 MHz and ^{13}C NMR at 126 MHz on Varian spectrometers in D_2O or D_2O with addition of CD_3CN. Chemical shifts reported in δ (ppm) are referenced to external acetone (δ_H = 2.225 ppm, δ_C =31.07 ppm).

N^1,N^3-bis{2-[bis(2-((2-Aminoethyl)amino)-2-oxoethyl)amino)ethyl)-2-(2-(2-(2-(prop-2-yn-1-yloxy)ethoxy)ethoxy]ethyl}malonamide, 1

Boc dendrimer **10** (12. 2 mg, 0.01 mmol) was treated with TFA (1 mL). TLC after 0.5 h indicated the Boc residue was cleaved. The solution was concentrated, dissolved in water and lyophilized. ^{1}H NMR (D_2O, 600MHz): δ = 4.21 (s, 2 H, $CH_{2\,propargyl}$), 4.04 (s, 8 H, $CH_2NH_3^+$), 3.70 - 3.74 (m, 2 H, CH_2O), 3.66 - 3.69 (m, 2 H, CH_2O), 3.65 (d, *J*=5.5 Hz, 2 H, CH_2O), 3.62 (d, *J*=5.5 Hz, 2 H, CH_2O), 3.59 – 3.52 (m,13 H), 3.43 - 3.52 (m, 4 H), 3.27 - 3.34 (m, 4 H, CH_2N), 3.14 (t, *J*=6.0 Hz, 8 H, CH_2N), 2.88(t, *J*=2.4 Hz, 1 H, $CH_{propargyl}$), 2.13 ppm (dd, 2 H, *J*=7.0 Hz, *J*=13.5 Hz, CH_2). ^{13}C NMR (126MHz, D_2O): δ = 173.2 (*C*O), 170.8 (*C*O), 163.9 (*C*O_{TFA}), 118.5 (*C*F_{TFA}), 116.2 (*C*F_{TFA}),, 80.3 (*C*$CH_{propargyl}$), 77.0(C*C*$H_{propargyl}$), 70.5 (O*C*H_2), 70.4 (O*C*H_2), 70.3 (O*C*H_2), 69.7 (O*C*H_2), 68.9 (O*C*H_2), 58.9 (O*C*$H_{2\,propargyl}$), 57.6 (N*C*H_2), 56.2 (N*C*H_2),, 51.3(*C*H), 39.9(N*C*H_2),, 37.8(N*C*H_2),, 37.1(N*C*H_2),, 30.2 ppm(*C*H_2). HRMS (ESI) Calcd. for $[M + H]^+$ $C_{32}H_{63}N_{12}O_9$: 759.4835. Found: 759.4856.

2-{2-(2-(2-azidoethoxy)ethoxy)ethyl)-N^1,N^3-bis(2-(bis(2-((2-aminoethyl)amino)-2-oxoethyl)amino)ethyl)malonamide 2

TFA (1 mL, as solvent) was added dropwise to the compound **18** (0.005 g, 0.004 mmol) at 0 ^{o}C and then the reaction was left for 2 h at room temperature. The mixture was stirred at room temperature until the TLC showed complete reaction of starting material. The solution was concentrated in vacuo and co-evaporated several times adding CH_2Cl_2-toluene to remove residual TFA to give the product in quantitative yield. This nearly pure material was directly used for subsequent reaction: HRMS (ESI): m/z calcd for $C_{29}H_{59}N_{15}O_8Na$ [M+Na]+: 768.4563, found: 768.4548.

3-{2-[2-(2-chloroethoxy) ethoxy] ethoxy} prop-1-yne, 6

To a solution of 2-[2-(2-chloroethoxy) ethoxy] ethanol, **5**, (10 g, 59.30 mmol) and propargyl bromide (7.05 g, 59.30 mmol) in DMF (50 mL), NaH (2.4 g, 59.30 mmol, 60% in mineral oil) was added portionwise at 0 °C. The reaction mixture was allowed to warm to room temperature and stirred for 2 h. The reaction was quenched with H_2O (100 mL) and the mixture was extracted with EtOAc (2 x 20

mL). The combined organic layers were dried over anhydrous Na_2SO_4 and the solvent was removed under reduced pressure to afford **6** (11.2 g, 91%) as an oil which was taken to the next reaction without further purification: R_f = 0.49 (EtOAc/hexane 3:1); ^{1}H NMR (500 MHz, $CDCl_3$): δ 4.20 (d, 2H, J = 2.4 Hz, OCH_2-C≡), 3.76 (t, 2H, J = 6.0 Hz, CH_2), 3.72–3.65 (m, 8H, CH_2x 4), 3.63 (t, 2H, J = 6.0 Hz, CH_2), 2.43 (t, 1H, J = 2.4 Hz, ≡C-H); ^{13}C NMR (176 MHz, $CDCl_3$) δ 79.6 (≡CCH$_2$), 77.2 (≡CH), 77.03 (CH$_2$), 76.85 (CH$_2$), 74.50 (CH$_2$), 71.35 (CH$_2$), 70.64 (CH$_2$), 70.61 (CH$_2$), 70.47 (CH$_2$), 69.10 (CH$_2$), 58.39 (CH$_2$), 42.70 (CH$_2$); IR cm^{-1} 3290.9 (≡CH), 2115.8 (C≡C). HRMS (ESI) Calcd. for (M + Na)$^+$ $C_9H_{15}NaO_3$: 229.0602. Found: 229.0601.

Diethyl 2-{2-[2-(2-(prop-2-yn-1-yloxy)ethoxy)ethoxy]ethyl}malonate, 7

To a solution of diethylmalonate (14.64 mL, 96.8 mmol) in anhydrous THF (75 mL) was added NaH (3.9 g, 96.8 mmol, 60% in mineral oil) portionwise at 0 °C and the reaction was then allowed for 30 mins at room temperature until clear solution is formed. A solution of **6** (10 g, 48.39 mmol) in anhydrous THF (30 mL) was added dropwise and the reaction mixture was stirred at room temperature overnight. The solid was removed from the solution via filtration and the resulting clear solution was concentrated under reduced pressure. The crude product was purified with column chromatography (EtOAc/hexane 3:1) to yield **7** (14.1 g, 88%) as an oil: R_f = 0.48 (EtOAc/hexane 3:1); ^{1}H NMR (500 MHz, $CDCl_3$): δ 4.30 (qd, 4H, J = 7.1, 4.0 Hz, CH_2CH$_3$ x 2), 4.21 (d, 2H, J = 2.5 Hz, ≡CCH_2O), 3.72–3.69 (m, 1H), 3.69–3.66 (m, 2H, CH_2), 3.65–3.61 (m, 2H, CH_2), 3.60–3.56 (m, 2H, CH_2), 3.56–3.50 (m, 3H, CH_2, CH), 2.43 (t, 1H, J = 2.5 Hz, H-C≡), 2.18 (dt, 2H, J = 7.3, 6.1 Hz, CH_2), 1.27 (t, 6H, J = 7.1 Hz, CH_3 x 2); ^{13}C NMR (125 MHz, $CDCl_3$) δ 169.3 (CO), 79.7 (≡CCH$_2$), 74.5 (≡CH), 70.5 (CH$_2$), 70.4 (CH$_2$), 70.2 (CH$_2$), 69.1 (CH$_2$), 68.4 (CH$_2$), 61.3 (CH$_2$), 58.4 (CH$_2$), 48.9 (CH), 28.8 (CH$_2$), 14.1 (CH$_3$); IR cm^{-1} 3271.58 (≡CH), 2117.6 (C≡C), 1731.09 (CO). HRMS (ESI) Calcd. for (M + NH$_4$)$^+$ $C_{16}H_{30}NO_7$: 348.2017. Found: 348.2018.

N^1,N^3-bis(2-Aminoethyl)-2-{2-[2-(2-(prop-2-yn-1-yloxy) ethoxy) ethoxy] ethyl} malonamide, 8

A solution of **7** (4 g, 12.11 mmol) in freshly distilled ethylene diamine (33.6 mL, 0.51 mol) was heated at 50 °C for 48 hrs. The excess ethylene diamine was then co-evaporated with a mixture of toluene/methanol (3:1) (4 x 50 mL) and the resulting semisolid mass was triturated with diethyl ether to give crude **8** (4.03 g, 93%) as an off-white semi-solid contaminated with ethylene diamine which used directly in the next step.

Dimethyl 3,13-bis(2-methoxy-2-oxoethyl)-7,9-dioxo-8-{2-[2-(2-(prop-2-yn-1-yloxy)ethoxy)ethoxy]ethyl}-3,6,10,13-tetraazapentadecane-1,15-dioate, 9

To a solution of **8** (2 g, 5.6 mmol) in dry acetonitrile (50 mL) was added anhydrous Na_2CO_3 (2.4 g, 22.3 mmol) and the reaction mixture was allowed for 30 min at room temperature. Methyl bromoacetate (3.54 g, 2.2 mL, 23.2 mmol) was added dropwise and the reaction mixture was heated at 60 °C for 2 hr. The mixture was then cooled to room temperature, the solid was filtered and the solvent was removed under reduced pressure. The resulting crud mass was purified with column chromatography (EtOAc/CH_3OH 15:1) affording **9** (3.2 g, 88%) as a yellowish syrup: R_f = 0.45 (EtOAc/CH_3OH 15:1); ^1H NMR (500 MHz, $CDCl_3$): δ 7.53 (t, 2H, J = 5.0 Hz, NH_2), 4.22 (d, 2H, J = 2.4 Hz, ≡CCH_2O), 3.73 (s, 12H, CH_3 x 4), 3.71 (dd, 2H, J = 3.8, 1.8 Hz, CH_2), 3.69 (dd, 2H, J = 3.6, 1.8 Hz, CH_2), 3.64 (dd, 2H, J = 5.9, 3.3 Hz, CH_2), 3.60 (dd, 2H, J = 5.9, 3.3 Hz, CH_2), 3.58 (s, 8H, NCH_2CO x 4), 3.53 (t, 2H, J = 6.2 Hz, CH_2), 3.38–3.25 (m, 5H, CONHCH_2 x 2, C*H*), 2.89 (t, 4H, J = 6.1 Hz, CH_2N x 2), 2.47 (t, 1H, J = 2.4 Hz, *H*-C≡), 2.17 (q, 2H, J = 6.3 Hz, CH_2); ^{13}C NMR (125 MHz, $CDCl_3$) δ 171.9 (CO), 170.6 (CO), 79.6 (≡*C*CH_2), 74.7 (≡*C*H), 70.5 (*C*H_2), 70.3 (*C*H_2), 70.0 (*C*H_2), 69.1 (*C*H_2), 68.5 (*C*H_2), 58.3 (*C*H_2), 54.9 (CONH<u>*C*</u>H_2), 53.1 (<u>*C*</u>H_2N), 51.7 (*C*H_3O), 51.2 (*C*H), 37.6 (N<u>*C*</u>H_2CO), 31.6 (*C*H_2); IR cm^{-1} 3302.9 (≡CH), 1739.3 (CO), 1669.7 (CO). HRMS (ESI) Calcd. for $(M + Na)^+$ $C_{28}H_{46}N_4NaO_{13}$: 669.2954. Found: 669.244.

N[1],N[3]-bis{2-[bis(2-((2-tert-butyl-carbamidoethyl)amino)-2-oxoethyl)amino)ethyl)-2-(2-(2-(2-(prop-2-ynyloxy)ethoxy)ethoxy]ethyl}malonamide 10

A solution of **9** (1 g, 1.5 mmol) in freshly distilled ethylene diamine (5 mL, 56.8 mmol) was heated at 50 °C for 3 days. The excess ethylene diamine was then coevaporated with a mixture of toluene/methanol (3:1) (4 x 10 mL) and the resulting semisolid mass was triturated with diethyl ether several times to give crude **1.** The crude mixture of **1** (48 mg) was dissolved in methanol (3.5 mL) and Boc anhydride (290 mg) was added followed by triethylamine (185 μL). After stirring for 0.5 h TLC (DCM/MeOH 10:1 and 5:1) indicated the reaction was complete. The mixture was concentrated then dissolved in DCM and chromatographed on silica gel column using a DCM/MeOH gradient from 5% to 20% MeOH. The first fraction contained di-Boc derivative of ethylenediamine (18 mg), the second Boc dendrimer **10**. This was concentrated and dissolved in water/acetonitrile and lyophilized to provide a white solid product (49 mg). It was further purified on preparative RP HPLC C18 Luna(2) using a water acetonitrile gradient (A: H_2O + 0.1% CH_3CN, B: CH_3CN). Fractions containing the dendrimer were combined and lyophilized to afford a white fluffy powder (42.6 mg). R_f = 0.57 (DCM/CH_3OH 10:1); ^1H NMR (700MHz, D_2O): δ = 4.20 (s, 2 H, C$H_{2\ propargyl}$), 3.69 - 3.73 (m, 2 H, OCH_2), 3.64 - 3.67 (m, 2 H, OCH_2) , 3.61 - 3.64 (m, 2 H, OCH_2), 3.58 - 3.61 (m, 2 H, OCH_2), 3.47 - 3.52 (m, 2 H, OCH_2), 3.35 - 3.39 (m, 1 H, C*H*), 3.21 - 3.34 (m, 20 H) NCH_2, 3.14 - 3.20 (m, 8 H, NCH_2), 2.85 - 2.87 (m, 1 H, C$H_{propargyl}$), 2.63 - 2.71 (m, 4 H, NCH_2), 2.05 - 2.13 (m, 2 H, CCH_2), 1.37 ppm (s, 36 H, CH_3), ^{13}C NMR (126MHz, D_2O/CD_3CN 7:2): δ = 174.3 (*C*O), 172.5 *C*O), 158.9, (*C*O), 81.6 (O*C*C_3), 70.8 (O*C*H_2), 70.6 (O*C*H_2), 69.9 (O*C*H_2), 69.2 (O*C*H_2), 59.4 (N*C*H_2), 59.0 (N*C*H_2), 55.2(N*C*H_2), 51.8 (*C*H), 40.5 (N*C*H_2), 40.2 (N*C*H_2), 38.5 (N*C*H_2), 30.7 (C*C*H_2), 28.9 (*C*H_3) ppm. HRMS (ESI) Calcd. for $[M + H]^+$ $C_{52}H_{95}N_{12}O_{17}$: 1159.6933. Found: 1159.6918.

2-(2-(2-(benzyloxy)ethoxy)ethoxy)ethanol 11

Benzyl ether **11** was prepared as previously described.[1]

^{1}H NMR (700 MHz, $CDCl_3$): δ 7.28 - 7.35 (m, 4 H, ArH), 7.21 - 7.28 (m, 1 H, ArH), 4.54 (s, 2 H, -C$\underline{H}_{2g}$), 3.67 - 3.71 (m, a H, -C$\underline{H}_{2a}$), 3.62 - 3.67 (m, 6 H, -C$\underline{H}_{2f}$, -C$\underline{H}_{2c}$, -C$\underline{H}_{2d}$), 3.59 - 3.62 (m, 2 H, -C$\underline{H}_{2e}$), 3.56 - 3.59 (m, 2 H, -C$\underline{H}_{2d}$), 2.64 - 2.71 (m, 1 -OH); ^{13}C NMR (176 MHz, $CDCl_3$): δ 138.1, 128.3, 127.7, 127.6, 73.2, 72.5, 70.7, 70.6, 70.4, 69.4, 61.7 ppm.

2-(2-(2-(benzyloxy)ethoxy)ethoxy)ethyl 4-methylbenzenesulfonate 12

p-toluenesulfonyl chloride (4.76 g, 24.9 mmol) was added portion wise to a stirred solution of **11** (5.0 g, 20.8 mmol) in anhydrous CH_2Cl_2 (20 mL) containing Et_3N (3.46 mL, 24.98 mmol) at 0 ^oC and then the reaction was left for 2 h at room temperature. The reaction mixture was quenched with ice/water (100 ml) and extracted with EtOAc (3 x 25 mL). The combined organic layers were dried over anhydrous Na_2SO_4. After removal of solvent the crude product was purified by column chromatography (ethyl acetate – hexane gradient elution) to afford **12** (7.08 g, 91.7%) as yellowish oil: R*f* = 0.5 (ethyl acetate/hexane, 1/4, v/v); ^{1}H NMR (700 MHz, $CDCl_3$): δ 7.76 - 7.79 (m, 2 H, ArH), 7.29 - 7.32 (m, 6 H, ArH), 7.25 - 7.27 (m, 1 H, ArH), 4.54 (s, 2 H, -C$\underline{H}_{2g}$), 4.12 - 4.15 (m, 2 H, -C$\underline{H}_{2a}$), 3.66 - 3.68 (m, 2 H -C$\underline{H}_{2b}$), 3.61 - 3.63 (m, 2 H, -C$\underline{H}_{2f}$), 3.58 - 3.60 (m, 2 H, -C$\underline{H}_{2e}$), 3.57 - 3.58 (m, 4 H, -C$\underline{H}_{2c}$, -C$\underline{H}_{2d}$), 2.41 (s, 3 H, -C$\underline{H}_3$); ^{13}C NMR (176 MHz, $CDCl_3$): δ 144.7, 138.2, 133.0, 129.8, 128.3, 127.9, 127.7, 127.6, 73.2, 70.8, 70.7, 70.6, 69.4, 69.2, 68.7, 21.6 ppm; HRMS (ESI): *m/z* calcd for $C_{20}H_{26}O_6SNa$ $[M+Na]^+$: 394.1450, found: 394.1456.

diethyl 2-(2-(2-(2-(benzyloxy)ethoxy)ethoxy)ethyl)malonate 13

To a solution of diethylmalonate (5.6 mL, 36.76 mmol) in anhydrous DMF (30 mL) was added NaH (1.47 g, 36.76 mmol, 60% in mineral oil) portion wise at 0 °C and the reaction was then allowed for 45 mins at room temperature. A solution of **12** (6.9 g, 18.59 mmol) in anhydrous DMF (15 mL) was added dropwise at room temperature and the reaction mixture was stirred overnight at 55°C. The solid was removed from the solution via filtration and the resulting clear solution was concentrated under reduced pressure. The crude product was

purified by column chromatography (ethyl acetate – hexane gradient elution) to yield **13** (5.91 g, 83.2%) as a yellowish oil**:** R*f* = 0.3 (ethyl acetate/hexane, 3/2, v/v); $[\alpha]^{D\ 21}$ = -0.10 (*c* = 1.19, $CHCl_3$); ^{1}H NMR (500 MHz, $CDCl_3$): δ 7.31 - 7.37 (m, 4 H, ArH), 7.26 - 7.30 (m, 1 H, ArH), 4.57 (s, 2 H, -C$\underline{H}_{2g}$), 4.15 - 4.23 (m, 4 H, 2 × -C$\underline{H}_{2i}$), 3.66 - 3.70 (m, 2 H, -C$\underline{H}_{2e}$), 3.62 - 3.66 (m, 4 H, -C$\underline{H}_{2c}$, -C$\underline{H}_{2f}$), 3.56 - 3.60 (m, 2 H, -C$\underline{H}_{2d}$), 3.51 - 3.56 (m, 3 H, -C$\underline{H}_{2b}$, -C$\underline{H}_{h}$), 2.18 (dt, *J*=7.3, 6.1 Hz, 2 H, -C$\underline{H}_{2a}$), 1.24 - 1.28 (m, 6 H, 2 × -C$\underline{H}_{3j}$); ^{13}C NMR (126 MHz, $CDCl_3$): δ 169.4, 169.4, 138.3, 128.3, 127.7, 127.6, 73.2, 70.7, 70.6, 70.3, 69.5, 68.4, 61.3, 48.9, 28.8, 14.1 ppm; HRMS (ESI): m/z calcd for $C_{20}H_{30}O_7Na$ [M+Na]+: 405.1884, found: 405.1884.

N^1,N^3-bis(2-aminoethyl)-2-(2-(2-(2-(benzyloxy)ethoxy)ethoxy)ethyl)malonamide 14

A solution of **13** (5.0 g, 13.08 mmol) in freshly distilled ethylene diamine (20 mL) was heated at 50 °C for 48 hrs, at which point ESI-MS calcd for $C_{20}H_{34}N_4O_5$ [M + Na]$^+$ *m/z*, 433.25; found, 433.6 confirmed the complete conversion of amination of all four ester groups. The excess ethylene diamine was then co-evaporated with a mixture of toluene/methanol (3:1) (4 x 20 mL) and the resulting semisolid mass was triturated with diethyl ether to give **14** (4.56 g, 85.1%) as white solid. This crude material was used directly used for the next reaction.

Diethyl 8-(2-(2-(2-(benzyloxy)ethoxy)ethoxy)ethyl)-3,13-bis(2-ethoxy-2-oxoethyl)-7,9-dioxo-3,6,10,13-tetraazapentadecane-1,15-dioate 15

To a solution of **14** (1 g, 2.43 mmol) in dry acetonitrile (20 mL) was added anhydrous Na_2CO_3 (1.03 g, 9.75 mmol) and the reaction mixture was stirred for 45 min at room temperature. Ethyl bromoacetate (1.1 mL, 9.75 mmol) was added dropwise and the reaction mixture was heated at 60°C for 18 h. The mixture was then cooled to room temperature, the solid was filtered and the solvent was removed under reduced pressure. The crude product was purified with column chromatography (acetone – hexane gradient elution) to yield **15** (1.6 g, 87.1%) as yellow oil: R*f* = 0.3 (acetone /hexane, 6/1, v/v); ^{1}H NMR (500 MHz, $CDCl_3$): δ 7.52 (t, *J*=4.4 Hz, 2 H, 2 × -N$\underline{H}$), 7.30 - 7.34 (m, 4 H, ArH), 7.23 - 7.28 (m, 1 H, ArH), 4.53 - 4.56 (m, 2 H, -C$\underline{H}_{2g}$), 4.09 - 4.18 (m, 8 H, 4 × -C$\underline{H}_{2l}$), 3.64 - 3.67 (m,

2 H, -C$\underline{H}_{2e}$), 3.59 - 3.63 (m, 4 H, -C$\underline{H}_{2c}$, -C$\underline{H}_{2f}$), 3.54 - 3.58 (m, 2 H, -C$\underline{H}_{2d}$), 3.47 - 3.54 (m, 10 H, 4 × -C$\underline{H}_{2k}$, -C$\underline{H}_{2b}$), 3.24 - 3.31 (m, 5 H, 2 × -C$\underline{H}_{2i}$, -C$\underline{H}_{h}$), 2.84 (t, *J*=5.7 Hz, 4 H, 2 × -C$\underline{H}_{2j}$), 2.15 (q, *J*=6.4 Hz, 2 H, -C$\underline{H}_{2a}$), 1.22 - 1.28 (m, 11 H, 4 × -C$\underline{H}_{3m}$); ^{13}C NMR (126 MHz, $CDCl_3$): δ 171.5, 171.4, 170.5, 170.5, 138.2, 138.2, 128.3, 127.7, 127.6, 73.2, 70.6, 70.5, 70.1, 69.4, 68.5, 60.6, 55.1, 53.0, 51.2, 37.7, 31.6, 14.2, 14.2 ppm; HRMS (ESI): m/z calcd for $C_{36}H_{58}N_4O_{13}Na$ [M+Na]+: 777.3893, found: 777.3892.

2-(2-(2-(2-(benzyloxy)ethoxy)ethoxy)ethyl)-N^1,N^3-bis(2-(bis(2-((2-tert-butyl-carbamidoethyl)amino)-2-oxoethyl)amino)ethyl)malonamide 16

A solution of **15** (1.2 g, 1.59 mmol) in freshly distilled ethylene diamine (8 mL) was heated at 50 °C for 48 hrs, at which point ESI-MS calcd for $C_{36}H_{66}N_{12}O_9$ [M + Na]$^+$ *m/z*, 833.5; found, 833.78 confirmed the complete conversion of amination of all four groups. The excess ethylene diamine was then co-evaporated with a mixture of toluene/methanol (3:1) (4 x 10 mL) and the resulting semisolid mass was triturated with diethyl ether to give crude (1.11 g, 86.2%) as white solid. Magnetically stirred molten Boc_2O (1.07 g, 4.88 mmol) was added portion wise to a stirred solution of the white solid (0.90 g, 1.11 mmol) in anhydrous CH_2Cl_2 (10 mL) containing Et_3N (5 mL) at 0 ^{o}C and then the reaction was allowed to proceed for 2 hrs at room temperature and stirred overnight. The reaction mixture was extracted with CH_2Cl_2 (3 × 10 mL). The combined organic extracts were washed with 0.1 N aqueous HCl (3 × 10 mL) followed by saturated aqueous $NaHCO_3$ solution (1 × 50 mL). The organic phase was dried over $MgSO_4$ and the solvent was removed under reduced pressure. The crude product was purified by column chromatography (acetone – hexane gradient elution) to yield **16** (1.19 g, 88.7%) as a white solid**:** R*f* = 0.5 (acetone /hexane, 4/1, v/v); ^{1}H NMR (500 MHz, $CDCl_3$): δ 7.74 (br. s., 5 H, 5 × -N$\underline{H}$), 7.29 - 7.34 (m, 4 H, ArH), 7.23 - 7.28 (m, 1 H, ArH), 5.62 (br. s., 3 H, 2 × -N$\underline{H}$), 4.53 (s, 2 H, -C$\underline{H}_{2g}$), 3.56 - 3.66 (m, 8 H, -C$\underline{H}_{2c}$, -C$\underline{H}_{2f}$, -C$\underline{H}_{2e}$, -C$\underline{H}_{2d}$), 3.56 - 3.66 (m, 2 H, -C$\underline{H}_{2b}$), 3.46 - 3.50 (m, 1 H, -C$\underline{H}_{h}$), 3.07 - 3.43 (m, 28 H, 2 × -C$\underline{H}_{2i}$, 4 × -C$\underline{H}_{2k}$, 4 × -C$\underline{H}_{2l}$, 4 × -C$\underline{H}_{2m}$), 2.57 – 2.67 (m, 4 H, 2 × -C$\underline{H}_{2j}$), 2.17 – 2.27 (m, 2 H, -C$\underline{H}_{2a}$), 1.40 (s, 36 H, 4 ×[3 × -C$\underline{H}_{3n}$]); ^{13}C NMR (126 MHz, $CDCl_3$): δ 171.3, 156.7, 156.6, 137.9, 128.4, 127.8, 127.8, 79.3, 73.2, 70.5,

70.4, 70.1, 69.4, 68.6, 58.9, 55.0, 51.4, 40.4, 39.8, 37.2, 29.9, 28.5 ppm; HRMS (ESI): *m/z* calcd for $C_{56}H_{98}N_{12}O_{17}Na$ $[M+Na]^+$: 1233.7065, found: 1233.7066.

N^1,N^3-bis(2-(bis(2-((2-tert-butyl-carbamidoethyl)amino)-2-oxoethyl)amino)ethyl)-2-(2-(2-(2-hydroxyethoxy)ethoxy)ethyl)malonamide 17

Compound **16** (0.5 g, 0.413 mmol) was dissolved in CH_2Cl_2 (15 mL), $Pd(OH)_2$ on carbon (20%, 0.090 g) was added. Then it was stirred under 1 atmosphere of hydrogen gas at 21 °C for 16 h. After filtration through a celite pad the pad was washed with CH_2Cl_2 (3 x 10 mL). The crude product was purified by column chromatography (acetone – hexane gradient elution) to yield **17** (0.425 g, 92.2%) as a white solid: R*f* = 0.4 (acetone /hexane, 3/1, v/v); ^{1}H NMR (500 MHz, $CDCl_3$): δ 7.80 (br. s., 5 H, 5 × -N<u>H</u>), 5.63 (br. s., 3 H, 2 × -N<u>H</u>), 3.75 - 3.78 (m, 2 H, -C<u>H</u>$_{2f}$), 3.58 - 3.66 (m, 7 H, -C<u>H</u>$_{2c}$, -C<u>H</u>$_{2d}$, -C<u>H</u>$_{2e}$, -C<u>H</u>$_h$), 3.56 - 3.58 (m, 2 H, -C<u>H</u>$_{2b}$), 3.11 - 3.46 (m, 28 H, 2 × -C<u>H</u>$_{2i}$, 4 × -C<u>H</u>$_{2k}$, 4 × -C<u>H</u>$_{2l}$, 4 × -C<u>H</u>$_{2m}$), 2.66 (t, *J*=5.4 Hz, 4 H, 2 × -C<u>H</u>$_{2j}$), 2.19 – 2.23 (m, 2 H, -C<u>H</u>$_{2a}$), 1.42 (s, 36 H, 4 ×[3 × -C<u>H</u>$_{3n}$]); ^{13}C NMR (126 MHz, $CDCl_3$): δ 171.4, 156.8, 79.4, 72.5, 70.0, 68.3, 61.4, 59.0, 55.0, 51.0, 40.5, 39.8, 37.3, 30.2, 28.5 ppm**;** HRMS (ESI): m/z calcd for $C_{49}H_{92}N_{12}O_{17}Na$ $[M+Na]^+$: 1143.6596, found: 1143.6594.

2-(2-(2-(2-azidoethoxy)ethoxy)ethyl)-N^1,N^3-bis(2-(bis(2-((2-tert-butyl-carbamidoethyl)amino)-2-oxoethyl)amino)ethyl)malonamide 18

Mesyl chloride (0.043 g, 0.299 mmol) was added dropwise to a stirred solution of **17** (0.28 g, 0.249 mmol) in anhydrous CH_2Cl_2 (8 mL) containing Et_3N (0.04 mL, 0.299 mmol) at 0 °C and then the reaction was left for 2 hrs at room temperature. The reaction mixture was quenched with ice/water (10 ml) and extracted with EtOAc (3 x 5 mL). The combined organic layers were dried over anhydrous Na_2SO_4. Solvent removal under reduced pressure afforded the mesylated product (0.271 g, 90.6%) as a yellowish oil. A solution of the mesylate (0.32 g, 0.266 mmol) in DMF (6 mL) containing sodium azide (0.026 g, 0.4 mmol) was stirred for 1 h at room temperature and then the reaction was heated at 55 °C for 2 hrs. The mixture was then poured into ice-cold 0.5~ HCl and extracted with ether, the extract was washed with saturated aqueous $NaHCO_3$ solution (1 × 5

mL). The organic phase was dried with $MgSO_4$ and the solvent was removed under reduced pressure. The crude product was purified by column chromatography (acetone – hexane gradient elution) to yield **18** (0.273 g, 89.3%) as a white solid: R*f* = 0.45 (acetone /hexane, 4/1, v/v); ^{1}H NMR (500 MHz, $CDCl_3$): δ 7.74 (br. s., 5 H, 5 × -N$\underline{H}$), 5.60 (br. s., 3 H, 2 × -N$\underline{H}$), 3.59 - 3.67 (m, 6 H, -C$\underline{H}_{2c}$, -C$\underline{H}_{2d}$, -C$\underline{H}_{2e}$), 3.55 - 3.58 (m, 2 H, -C$\underline{H}_{2f}$), 3.50 (t, *J*=7.3 Hz, 2 H, -C$\underline{H}_{2b}$), 3.36 - 3.46 (m, 7 H, -C$\underline{H}_{h}$, 2 × -C$\underline{H}_{2i}$, -C$\underline{H}_{2k}$), 3.10 - 3.34 (m, 22 H, 3 × -C$\underline{H}_{2k}$, 4 × -C$\underline{H}_{2l}$, 4 × -C$\underline{H}_{2m}$), 2.64 – 2.66 (m, 4 H, 2 × -C$\underline{H}_{2j}$), 2.23 – 2.26 (m, 2 H, -C$\underline{H}_{2a}$), 1.41 (s, 36 H, 4 ×[3 × -C$\underline{H}_{3n}$]); ^{13}C NMR (126 MHz, $CDCl_3$): δ 171.3, 171.2, 156.8, 79.4, 70.5, 70.1, 69.9, 68.7, 58.9, 55.1, 51.4, 50.7, 40.4, 39.8, 37.2, 30.4, 28.5 ppm; IR cm^{-1} 2106.8 (N_3), 1665.4 (CO); HRMS (ESI): m/z calcd for $C_{49}H_{91}N_{15}O_{16}Na$ [M+Na]+: 1168.666, found: 1168.6672.

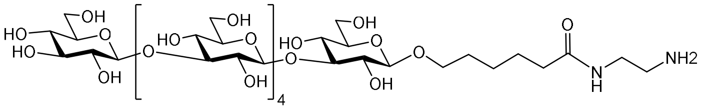

(2-Aminoethylamido)carbonylpentyl β-D-glucopyranosyl-(1→3)-β-D-glucopyranosyl- β-D-glucopyranosyl-(1→3)-β-D-glucopyranosyl- β-D-glucopyranosyl-(1→3)-β-D-glucopyranoside 19

A solution of 5-methoxycarbonylpentyl β-D-glucopyranosyl-(1→3)-β-D-glucopyranosyl-(1→3)-β-D-glucopyranosyl-(1→3)-β-D-glucopyranosyl-β-D-glucopyranosyl-(1→3)-β-D-glucopyranoside **3**[1] (11.5 mg, 10.3 μmol) in 1,2-diaminoethane (1mL) was stirred at 50 °C overnight.[2,3] After 23 h TLC (DCM/MeOH/H2O/AcOH 3:3:1:0.1) indicated the reaction was complete. The reaction mixture was concentrated and co evaporated with toluene. The residue was dissolved in water, neutralized with acetic acid and injected on a C18 semi preparative HPLC column (99A:1B 40g6 60A:40B, A: water + 0.1% AcOH, B: acetonitrile + 0.1% AcOH, t_R = 12.6 min, λ = 212 nm). Fractions containing the product were combined and lyophilized to provide **19** as a white solid (9.6 mg, 81%). R_f: 0.11 (DCM/MeOH/H2O/AcOH 3:3:1:0.1), ^{1}H NMR (600MHz, D_2O, 5 ^{o}C): δ = 4.70 - 4.79 (m, 5 H, 5 x H-1), 4.44 (d, 1 H, $J_{1,2}$= 8.1 Hz, H-1), 3.84 - 3.93 (m, 7 H), 3.60 - 3.76 (m, 12 H), 3.38 - 3.53 (m, 19 H), 3.40 - 3.35 (m, 1 H,), 3.31

(dd, 1 H, J = 8 Hz, J = 9.3 Hz), 3.05 (t, 2 H, J = 6.1 Hz, H_g), 2.26 (t, 2 H, J = 7.5 Hz, H_e), 1.64 - 1.57 (m, 4 H, H_b, H_d), 1.30 - 1.38 ppm (m, 2 H, H_c). ^{13}C NMR (176MHz, D_2O): δ = 177.4 (CO), 103.6(C-1), 103.3(C-1), 102.7(C-1), 85.3, 85.3, 85.1, 85.1, 84.9, 76.9, 76.8, 76.5, 76.4, 76.4, 74.3, 74.1, 73.7, 71.2 (OCH_2), 70.4, 69.0 61.5(*C*-6), 40.1 (NCH_2), 38.2 (NCH_2), 36.4 (CH_2), 29.2(CH_2), 25.6(CH_2), 25.5ppm (CH_2). HRMS (ESI) Calcd. for $[M+H]^+$ $C_{44}H_{79}N_2O_{32}$: 1147.461. Found: 1147.4629.

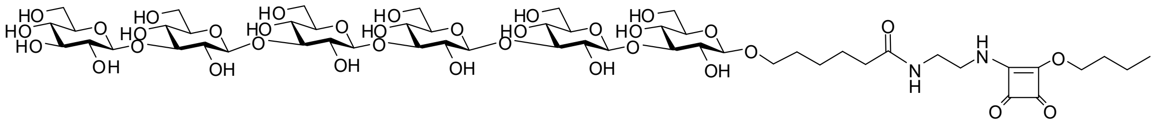

1-[(2-Aminoethylamido)carbonylpentyl β-D-glucopyranosyl-(1→3)-β-D-glucopyranosyl-(1→3)-β-D-glucopyranosyl-(1→3)-β-D-glucopyranosyl- β-D-glucopyranosyl-(1→3)-β-D-glucopyranoside]-2-butoxycyclobutene-3,4-dione 20

Amine **19** (9 mg, 7.8 μmol) was dissolved in water (0.35 mL) and ethanol (0.25 mL) was added to the solution. A solution of of 3,4-dibutoxy-3-cyclobutene-1,2-dione in ethanol (20%, 35 μL, 31.5 μmol) was added and the pH of the reaction mixture was adjusted to 8 by careful addition of $NaHCO_3$ solution[4,5]. TLC (DCM/MeOH/H2O/AcOH 3:3:1:0.1) after 0.5 h indicated the reaction was complete. The reaction mixture was acidified with 10% acetic acid and concentrated to remove ethanol then purified on a HPLC semi preparative column (C18) using a gradient of water-acetonitrile (95A:5B 45 g6 50A 50B, A: H2O + 0.02% AcOH, B: CH_3CH + 0.02% AcOH; t_R = 17.8 min, λ = 280 nm). Fractions containing the product were lyophilized to afford **20** as a white solid (9 mg, 89%). R_f = 0.83 (DCM/MeOH/H2O/AcOH 3:3:1:0.1), ^{1}H NMR (600MHz, D_2O, 5 °C): δ = 4.70 - 4.79 (m, 5 H, 5 x H-1), 4.62 - 4.69 (m, 2 H, H_h), 4.42 (d, J=7.9 Hz, 1 H, H-1), 3.89 – 3.87 (m, 7 H), 3.64 - 3.77 (m, 12 H), 3.54 - 3.62 (m, 2 H), 3.28 - 3.53 (m, 21 H), 2.13 - 2.22 (m, 2 H, H_e), 1.78 – 17.3 (m, 2 H, H_i), 1.45 - 1.60 (m, 4 H, H_b, H_d), 1.35 - 1.45 (m, 2 H, H_j), 1.31 – 1.25 (m, 2 H, H_c), 0.90 ppm (q, J=7.6 Hz, 3 H, H_k). ^{13}C NMR (126MHz, D_2O): δ = 189.7(CO), 184.2 (CO),

177.8 (C*C*O), 174.6 (C*C*N), 103.8 (C-1), 103.5 (C-1), 102.9(C-1), 85.4, 85.2, 85.1, 77.0, 76.6, 75.4, 74.4, 74.3, 73.9, 71.2, 70.6, 69.1 (C_a), 61.6 (C-6), 45.2 (C_g), 44.9, 40.4, 40.2 (C_f), 36.8 (C_e), 32.3 (C_b), 29.5 (C_i), 26.2 (C_d), 25.6 (C_c), 19.0 (C_j), 13.9 ppm (C_k). HRMS (ESI) Calcd. for $[M+Na]^+$ $C_{52}H_{86}N_2NaO_{35}$: 1321.4903. Found: 1321.494.

For NMR assignment atoms are labeled as follows

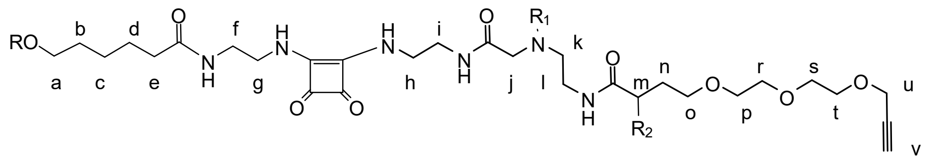

R: hexasacchride, R_1: dendrimer 1 arm, R_2: dendrimer 2 arms

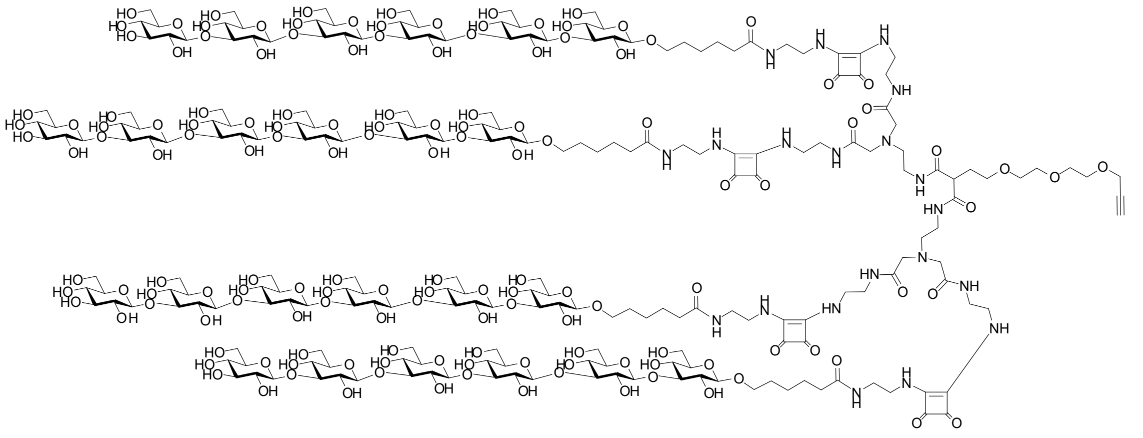

Hexasaccharide dendrimer 21

Dendrimer **1** (0.83 mg, 0.7 μmol) and squarate half ester **20** (5.45 mg, 4.2 μmol) were dissolved in 0.5 M borate buffer, pH: 9 (0.45 mL) and stirred at room temperature[5].The reaction progress was monitored by TLC and MALDI TOF MS. After 3 days the reaction mixture was acidified with acetic acid and then injected on a HPLC semi preparative C18 column (99A1B 50 g6 60A40B, A: H_2O + 0.02% AcOH, B: CH_3CN + 0.02% AcOH). The product of the reaction (t_R = 24.2 min, λ =280 nm) was eluted ahead of excess squarate (t_R =27.8 min, λ = 280 nm, 1.2 mg after lyophilization). The fraction containing hexasaccharide dendrimer **21** was lyophilized to afford a white solid (3.1 mg, 78%), R_f = 0.24 (DCM/MeOH/H2O/AcOH 3:3:1:0.1); ^1H NMR (600MHz, D_2O): δ = 4.68 - 4.78 (m, 20 H, 20 x H-1), 4.42 (d, *J*=8.1 Hz, 4 H, H-1), 4.20 (s, 2 H, CH_{2u}), 3.79 - 3.92 (m, 28 H), 3.56 - 3.79 (m, 66 H), 3.27 - 3.56 (m, 92 H), 3.22 (br. s., 12 H, CH_{2j} and CH_{2l}), 2.88 (t, 1 H, *J* = 2.4 Hz, CH_v), 2.56 - 2.66 (m, 4 H, CH_{2k}), 2.16 – 2.19 (m, 8 H, CH_{2e}), 2.00 - 2.09 (m, 2 H, CH_{2n}), 1.44 - 1.62 (m, 16 H, CH_{2b} and CH_{2d}), 1.27 - 1.29 ppm (m, 8 H, CH_{2c}). LC-UV-ESI-MS for $C_{224}H_{366}N_{20}O_{145}$: *m/z* calcd for $[M+3H]^{+3}$1886.7377 found 1886.7368; t_R = 11.7 min, λ = 280 nm (GlycanPac AXH-1, 0→2min, 10→20% B; 2→15 min, 20→45% B; 15→18 min, 45% B; A: 96% acetonitrile/ 4% 0.1 M ammonium formate, B: 0.1 M ammonium formate in H_2O pH: 4.4; flow rate 0.35 mL/min).

Hexasaccharide dendrimer 22

Dendrimer **2** (0.83 mg, 0.52 μmol) and squarate **20** (4.03 mg, 3.1 μmol) were dissolved in 0.5 M borate buffer pH 9 and left stirred at room temperature. The reaction progress was monitored by MALDI-TOF MS and LC/MS. After 48 h the reaction mixture was acidified with acetic acid and injected on C18 semi preparative HPLC column (99A1B 50 g6 60A40B, A: H_2O + 0.02% AcOH, B: CH_3CN + 0.02% AcOH; analytical C18 99A1B 35g6 60A40B, 22.7 min, λ = 280 nm). Product was separated from excess squarate (1.1 mg after lyophilisation). It was further purified on the same column (95A15B 30g6 60A40B, t_R = 22.5 min, λ = 280 nm) to give hexasaccharide dendrimer **22** as a white powder (2.1 mg, 72%). R_f: 0.21 (DCM-MeOH-H2O-AcOH = 3 : 3: 1 : 0.1) LC-UV-ESI-MS for

$C_{221}H_{363}N_{23}O_{144}$: *m/z* calcd for $[M+3H]^{+3}$1882.0669 found 1882.0686; t_R = 12.0min, λ = 280 nm (GlycanPac AXH-1, 0→2min, 10→20% B; 2→15 min, 20→45% B; 15→18 min, 45% B; A: 96% acetonitrile/ 4% 0.1 M ammonium formate, B: 0.1 M ammonium formate in H_2O pH: 4.4; flow rate 0.35 mL/min).

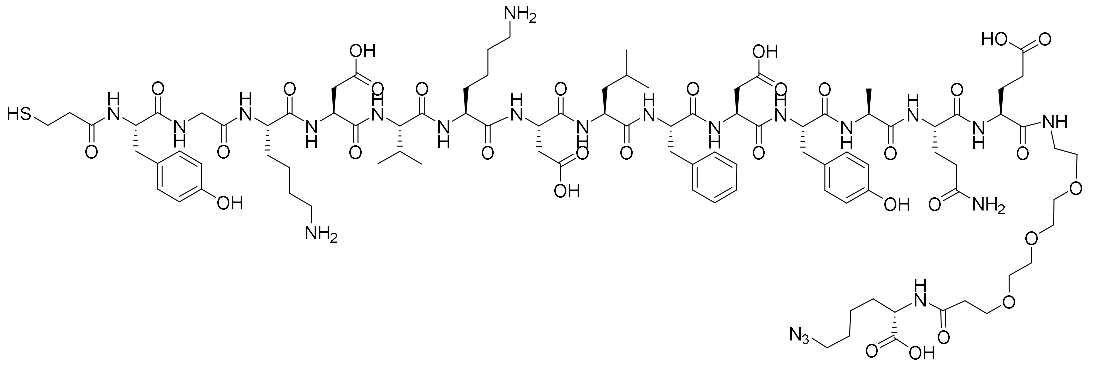

HS-$(CH_2)_2$CO-YGKDVKDLFDYAQE-tPeg-K(N_3)-OH 23

2-Chlorotrityl chloride resin (505 mg; 1.33 mmol/g) was swollen in DCM (4 mL) in a glass reactor and drained. A solution of Fmoc -Lys(N_3)-OH (62.5 mg, 0.158 mmol) in dry DCM (4 mL) with DIPEA (0.35 mL, 2 mmol) was added to the resin and it was gently agitated on a bench shaker for 0.5 h, then it was drained and washed twice with DMF. A solution of DCM/MeOH/DIPEA 80:15:5 was added to the resin and it was shaken for 10 min, drained and capping with MeOH was repeated. The resin was washed with DMF (5 mL x 3), drained, then deprotected with 25% piperidine in DMF (5 mL) for 5 min then drained and deprotection was repeated for 20min[6]. The resin was washed thoroughly with DMF and drained. A solution of Fmoc-tPeg-OH (133 mg, 0.3 mmol), HOBt (40 mg, 0.3 mmol), HBTU (109.4 mg, 0.29 mmol) in dry DMF (1.5 mL) with DIPEA (104.5 μL, 0.6 mL) was prepared and added to the resin and it was agitated for 1h then drained, washed 5 times with DMF and the coupling with Fmoc-tPeg-OH was repeated[6a]. The resin was washed with DMF, then with MeOH, hexane and stored under argon at -20 °C. Further couplings were performed on an automated peptide synthesizer ABI 433 A (Applied BioSystems). The resin was transferred to a large automated reaction vessel and swelled in NMP for 1h, drained and then automated synthesis was performed according to standard Fast-moc chemistry at 0.25 mmol scale starting with first step Fmoc deprotection then adding the next Fmoc

protected amino acid. The cycles were repeated until the last amino acid Fmoc-Tyr-OH was added, then after Fmoc deprotection Trt-$S(CH_2)_2CO_2H$ was added and the automated synthesis was complete. The resin was transferred to the glass reactor, washed with DMF and DCM then it was treated with a cleavage cocktail (10 mL, TFA/TIPS/H_2O 1:0.06:0.06) and left on a shaker. After agitating for 2 h the solution was drained and the resin was washed with TFA (10 mL x 3). The combined cleaving solution and the TFA washings were concentrated and co evaporated with toluene. The residue was precipitated with cold ethyl ether. The ether was removed by decantation and the precipitate was dissolved in degassed water with addition of acetonitrile and lyophilized to provide crude peptide **23** as a white fluffy solid (220 mg). It was purified by RP HPLC on preparative C18 column (72A28B 60 g6 55A 45B, A: water + 0.1% TFA, B: acetonitrile + 0.1% TFA, t_R = 23.7 min λ = 280 nm; 4 injections) to afford **23** as a fluffy white powder (53 mg, 17%). Analytical C18 (72A 28B 25 g6 65A 35B) t_R = 21.0 min, λ = 212 nm. LC-UV-ESI-MS for $C_{95}H_{142}N_{22}O_{32}S$: *m/z*: calcd for $[M+2H]^{2+}$1068.502, found1068.4997, t_R = 3.59 min, λ = 214 nm. MS/MS: *m/z* 376.22 (y2) 505.27 (y3), 633.31 (y4), 704.35 (y5), 867.39 (y6), 982.44 (y7), 1129.48 (y8), 1242.54 (y9), 1357.54 (y10), 1485.62 (y11), 1584.64 (y12),1699.6 (y13), 1827.81 (y14), 1884.92 (y15),; 437.19 (b3), 552.21 (b4), 651.27 (b5), 779.36 (b6), 894.37 (b7), 1007.45 (b8), 1154.49 (b9), 1269.50 (b10), 1432.54 (b11), 1503.60 (b12), 1631.60 (b13), 1760.60 (b14), 1963.78 (b15), 2135.85 $[M+H]^+$.

β-Man$_3$-$(CH_2)_3S(CH_2)_2NCO(CH_2CH_2O)_4COCH_2CH_2SCH_2CH_2CO$Fba-tPeg-K($N_3$) 4

Peptide **23** (7.4 mg, 3.46 μmol) in degassed water (1.2 mL) with acetonitrile (0.4 mL) was agitated until it dissolved. β-Man$_3$ acrylate **24** was prepared according to the procedure previously described[7]. Compound **24** (3.6 mg, 4.1 μmol) was dissolved in degassed 0.02M borate buffer pH 8.15 (0.3 mL) and added to the peptide solution[7,8]. The vial was rinsed with borate buffer (0.15 mL x 2) and added to the reaction mixture. The vial was purged with argon, wrapped in

aluminum foil and left on a shaker. The progress of the reaction was monitored by MALDI-TOF MS. After 5 h the reaction mixture was acidified with 10% acetic acid and injected on HPLC semi preparative column (2 injections, 80A:20B 50g6 60A:40B; A: water with 0.02 % AcOH, B: acetonitrile with 0.02 % AcOH). The first fraction contained excess acrylate **24** (t_R = 6 min; 1.3 mg after lyophilization). Fractions containing product were pooled (t_R = 30 min, λ = 212 nm) and lyophilized to afford glycopeptide **4** as a white solid (5.8 mg, 56%). Fraction at t_R = 42.8 min contained peptide disulfide dimer (1mg after lyophilisation). LC-UV-ESI-MS for **4** (Figure S1) $C_{130}H_{203}N_{23}O_{54}S_2$: *m/z* calcd for $[M+3H]^{+3}$ 1005.7835, found 1005.7835; (Fig.1), t_R = 7.4 min., λ = 214 nm (C18).

Conjugation of hexasaccharide dendrimer 21 and glycopeptide 4

Dendrimer **21** (2.98 mg, 0.53 μmol) and glycopeptide **4** (1.9 mg, 0.63 μmol) were dissolved in water in a 4 ml Kimball vial and lyophilized. Then a small stirring bar and copper powder (30 mg) were added. The vial was closed with a rubber septum and an open screw cap and the vial was degassed and filled with argon. Then degassed 0.2 M Tris buffer pH 8 was added (0.5 mL) and the suspension was degassed and purged with argon (5x) then bathophenantroline Cu^{+1} catalyst (25 μL) was added. The vial was wrapped in aluminum foil and left on a magnetic stirrer overnight. The reaction mixture was treated with 0.5 M EDTA pH 8 (0.9 mL), transferred to an Eppendorf tube and spun. The aliquot was taken into an Amicon Ultra-4 centrifugal filter unit (3,000 MWCO), dialyzed against degassed deionized water (4 mL x 3) and then concentrated. The concentrated solution was lyophilized to afford crude product as an off white solid (4.5 mg). LC/MS analysis employing a GlycanPac AXH-1 column revealed the presence of the expected product glycopeptide-hexasaccharide dendrimer **25** and excess substrate glycopeptide **4**. There was also a trace of glycopeptide with azide reduced to amine and some product as a result of ester hydrolysis of the "click" product. The product was purified on a GlycanPac AXH-1 analytical column employing multiple injections, (~ 0.5 mg per injection) of the crude product in the volume of 50 to 70 μL of the eluent, flow rate 1mL/min, applying linear gradient of solvent A and B (0→2 min, 10→20% A; 2→20 min, 20→35% A; 20→45 min,

35→60% A; 45→50 min, 60% A) where A was 0.1 M ammonium formate buffer pH 4.4 and B: 20% water and 80% acetonitrile. Fractions were checked by MALDI-TOF MS and those containing product **25** (t_R = 26.8 min., λ = 280nm) were combined and lyophilized. The lyophilized solid was dissolved in degassed water and lyophilized again. This was repeated several times to remove ammonium formate. Three batches of the product were obtained (1.54 mg total, 28%) as a white solid. Purity assessed by LC/MS ranged from 92 to 95% (Figure S1. LC-UV-ESI-MS (Figure S1) for $C_{354}H_{569}N_{43}O_{199}S_2$: *m/z* calcd for $[M+4H]^{+4}$ 2168.6365 found 2168.6295; t_R = 10.62 min, λ = 254 nm (0→2 min, 10→20% B; 2→20 min, 20→35% B; 20→25 min, 35→60% B; 25→28 min, 60% B) (Fig 2).

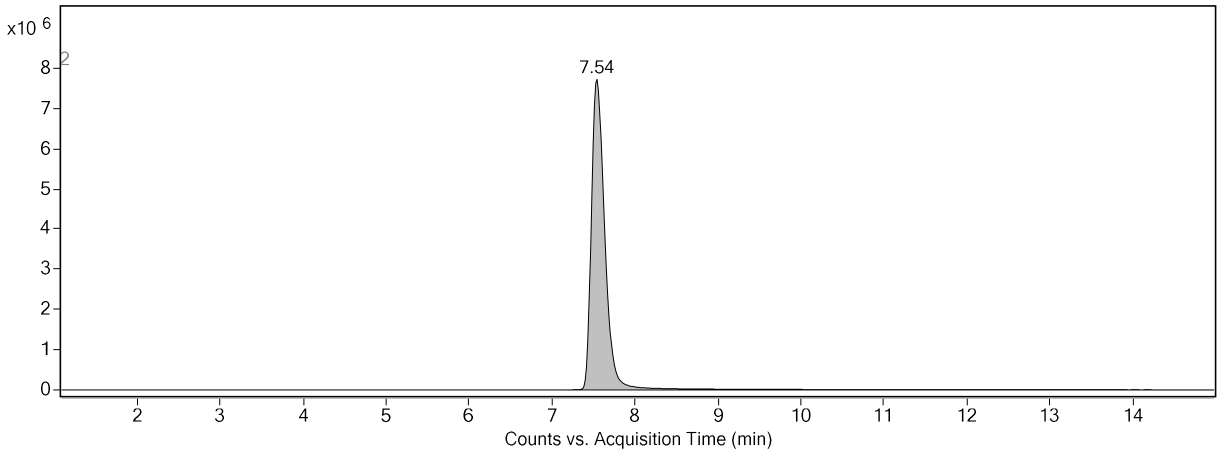

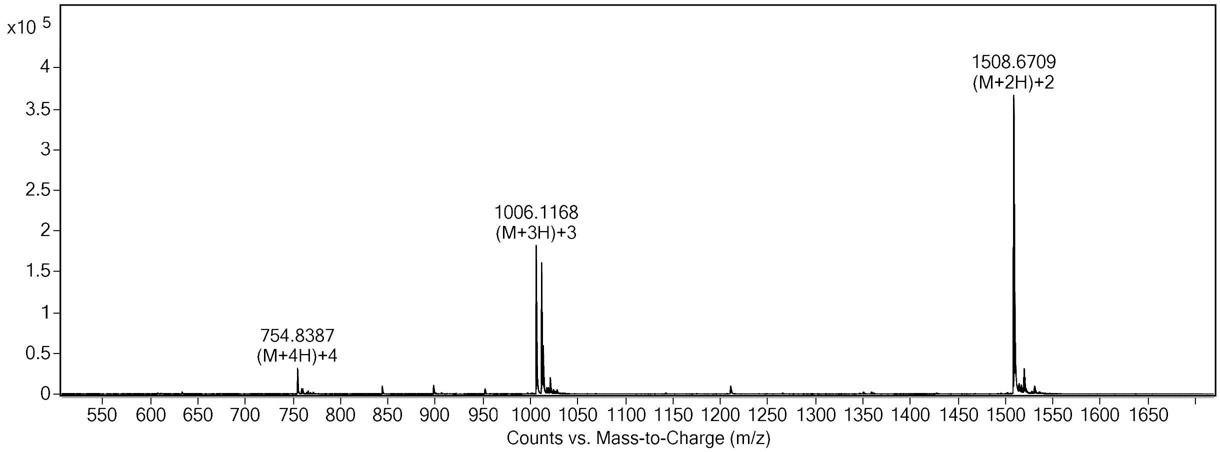

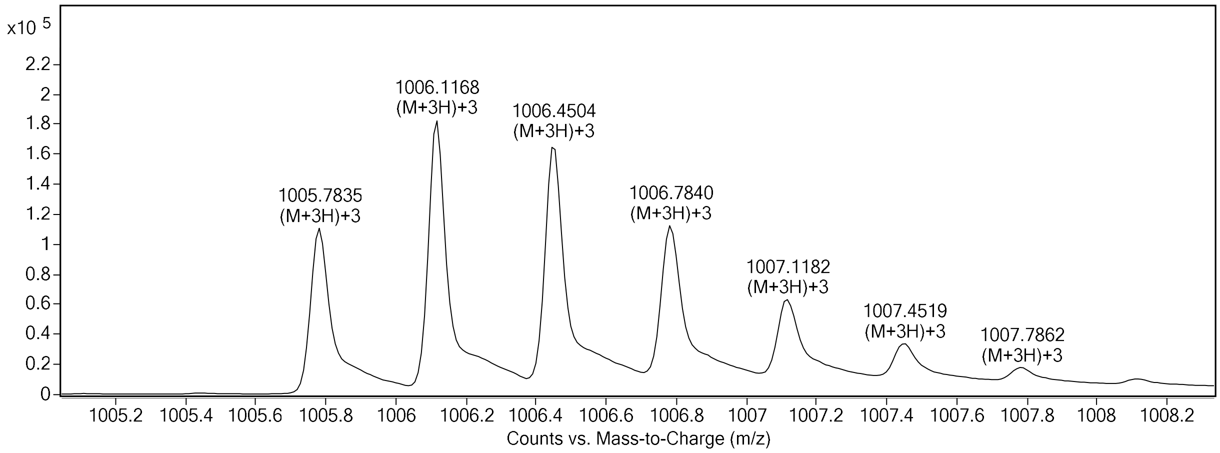

Figure S1. LC-UV-ESI-MS profile of compound **4**. Top graph:+ESI EIC; Middle: +ESI-MS; Bottom: +ESI-MS for $[M+3H]^{3+}$ (diff. 0.02 ppm)

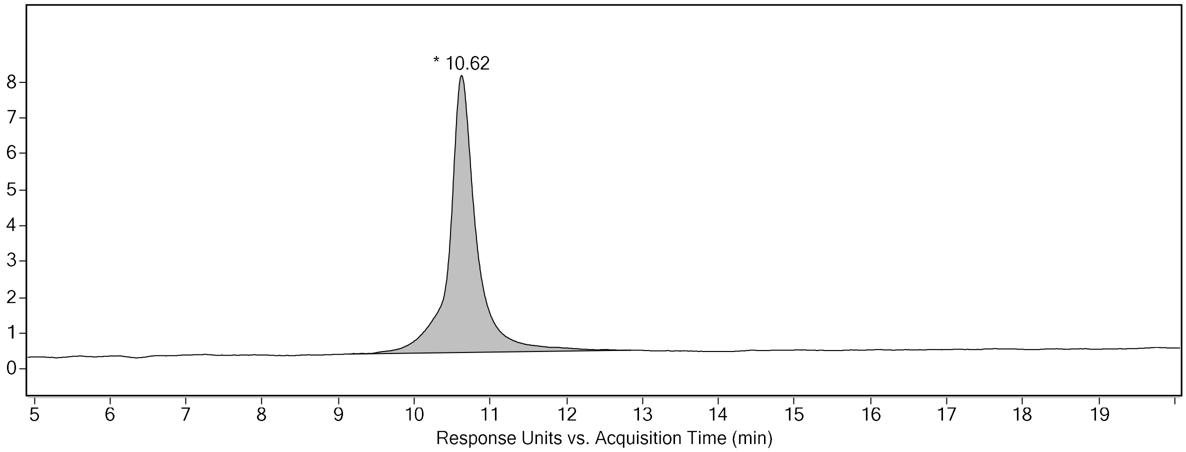

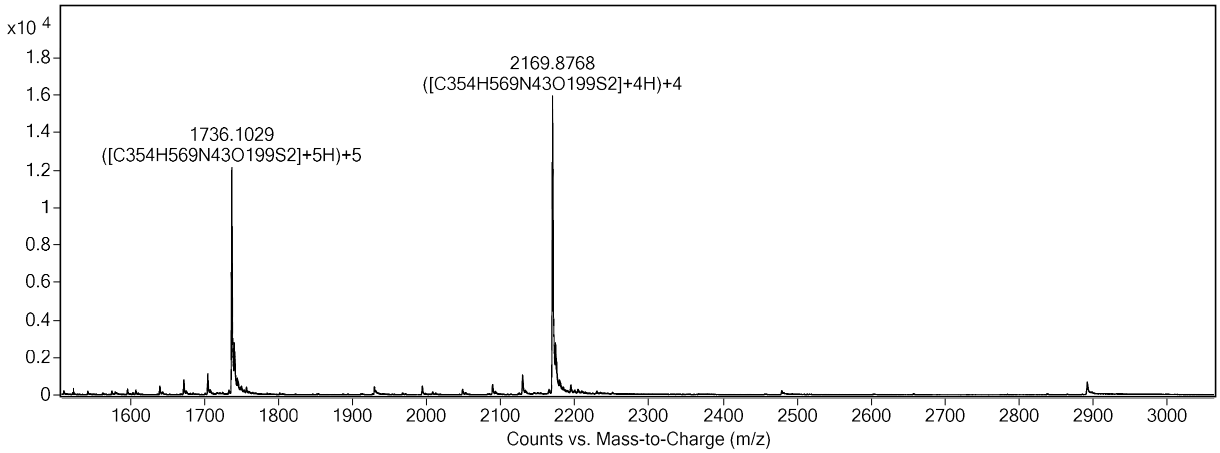

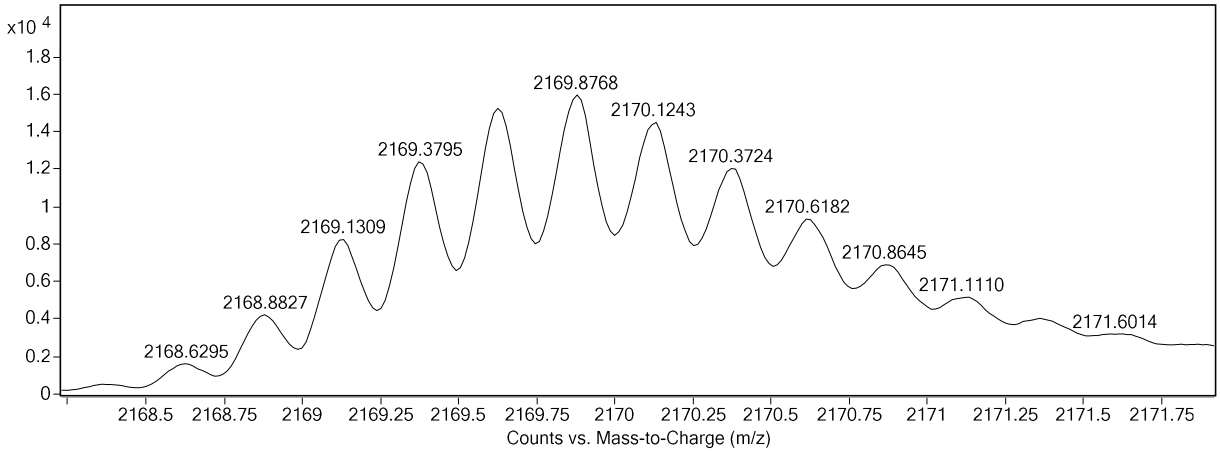

Figure S2. LC-UV-ESI-MS profile of compound **25**. Top graph: UV @ 254 nm; Middle: +ESI-MS; Bottom: +ESI-MS for $[M+4H]^{4+}$ (diff. 3.18 ppm)

Activation of Ovalbumin

Ovalbumin (2.22 μmol) and 3-prop-2-ynyloxy-propionic acid 2,5-dioxo-pyrrolidin-1-yl ester (6.66 μmol) were dissolved in 0.1 M PBS buffer pH 9 (600 μL) and stirred slowly at 21 ^{o}C for 1.5 days. Then, the reaction mixture was diluted with Milli-Q water (5 mL), filtered through Millipore filtration tube (10,000 MWCO, 4 x 10 mL), lyophilized and the ovalbumin-alkyne conjugate was obtained as a white foam. The MALDI-TOF mass spectrometry analysis indicated the conjugate had an average of 2 alkynes per Ovalbumin.

Conjugation of the dendrimer 22 to ovalbumin (Scheme S1)

Propargylated ovalbumin (3.02 mg, 0.068 μmol) and hexasaccharide dendrimer **22** (0.78 mg, 0.138 mmol) in 4 mL Kimball vial were dissolved in water and lyophilized. Then copper powder (~ 20 mg) and a small stirring bar were added. The vial was closed with a rubber septum and an open screw cap and purged with argon. Tris buffer 0.2 M pH 8 was added (0.3 mL) and the vial was degassed and purged with argon (5x). Cu^{+1} bathophenanthroline catalyst was added (25 μL) and the mixture was left on a stirring plate overnight. Next day the reaction mixture was treated with 0.5 M EDTA pH 8 (0.4 mL) and transferred to

an Eppendorf tube and spun then the solution was transferred to Amicon 4 mL centrifugal filter (10,000 MWCO) and dialysed against deionized water (4 x 4 mL). The concentrated solution was lyophilized to afford the product **26** as an off white solid (3.58 mg). MALDI-TOF MS indicated an average substitution of 1 dendrimer per molecule of ovalbumin.

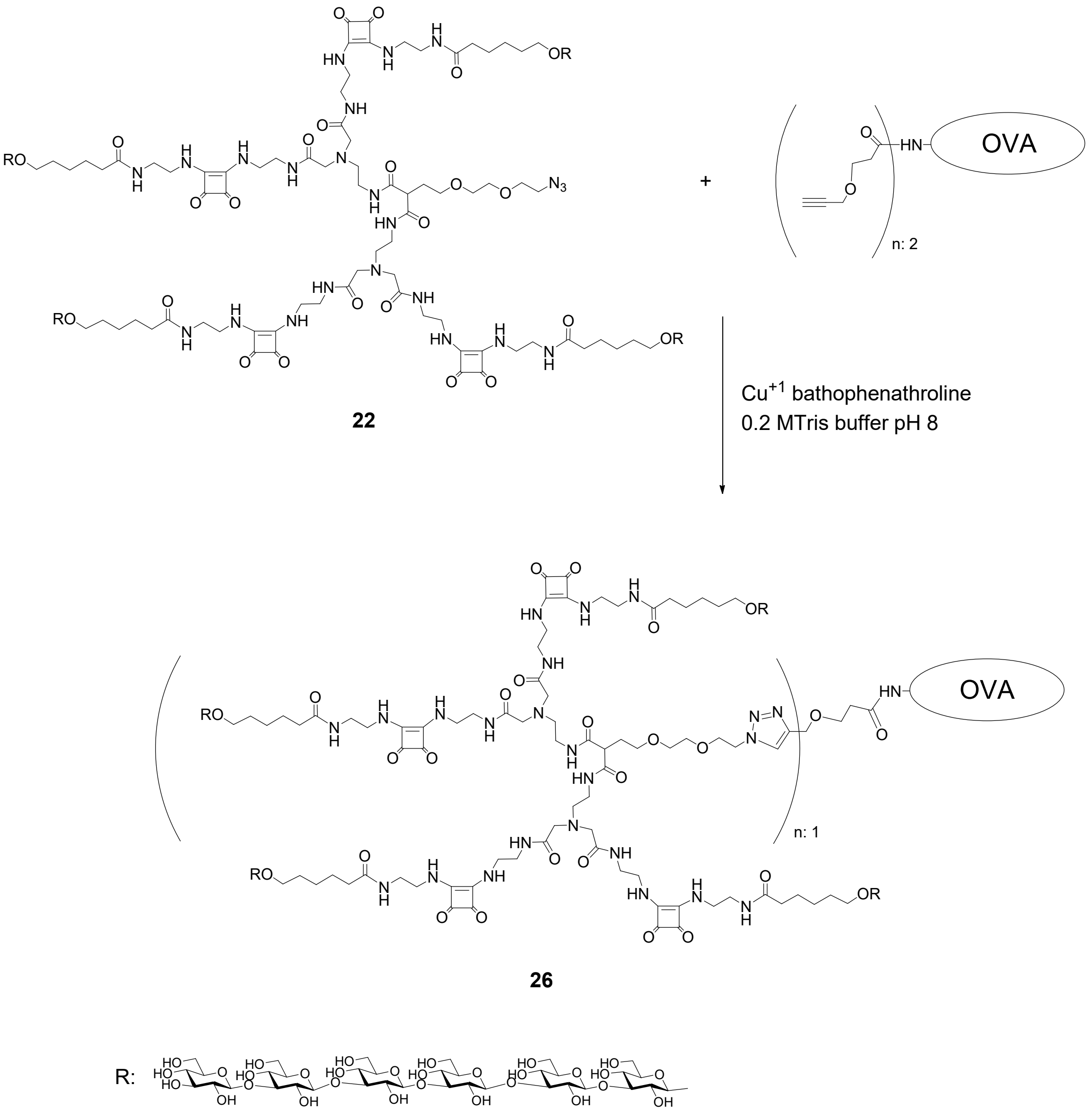

Scheme S1. Conjugation of dendrimer **22** to Ovalbumin previously activated by introduction of an alkyne group.

ELISA end point titers for mouse IgG

A: Dendrimer-beta glucan hexa-Fba-Man3 immunised mice:

OD *vs* dilution: Titration against Man_3 IgG

Dilution	Pre	D1	D2	D3	D4	D5	D6	D7	D8	D9	D10
100	0.1583	0.6805	2.4289	1.1162	0.9711	0.98	2.5501	1.7771	0.5277	1.1837	1.4926
316	0.0547	0.2317	1.6587	0.4494	0.3695	0.4376	1.7812	0.9472	0.1759	0.4472	0.6295
1000	0.0253	0.0662	0.6978	0.1417	0.1146	0.1392	0.767	0.3456	0.0492	0.1474	0.2237
3160	0.0024	0.0256	0.2187	0.0419	0.0413	0.0404	0.265	0.1047	0.0169	0.0565	0.0706
10000	-0.0022	0.0029	0.0581	0.0082	0.0088	0.0076	0.069	0.0275	0.0071	0.0351	0.0163
31600	-0.0012	0.0002	0.0173	0.0032	0.0024	0.0014	0.0235	0.0087	0.0022	0.0066	0.0071
100000	-0.0012	-0.0019	0.0071	-0.0001	0.0032	0.0003	0.0052	0.0005	-0.0003	0.0021	0.0011

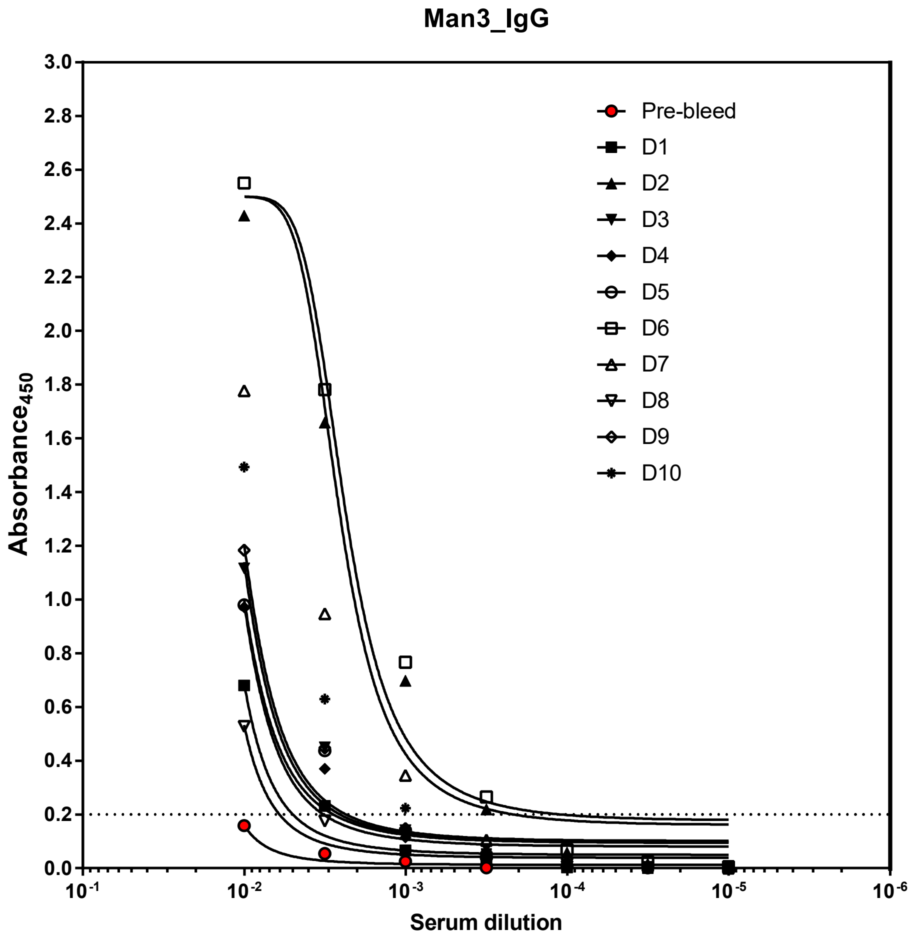

Figure S3

OD *vs* dilution: Titration curve against Fba IgG

Dilution	Pre	D1	D2	D3	D4	D5	D6	D7	D8	D9	D10
100	0.2312	0.7886	2.6034	1.1742	1.1821	1.2472	2.8443	2.1692	0.6859	1.5433	1.5896
316	0.0579	0.255	1.7734	0.4046	0.4165	0.4608	2.0211	1.0194	0.2111	0.5743	0.6316
1000	0.0175	0.0779	0.8048	0.1283	0.1391	0.1459	0.8652	0.3837	0.0665	0.222	0.2429
3160	0.0035	0.0209	0.2623	0.0427	0.0468	0.0461	0.312	0.1269	0.023	0.0809	0.078
10000	-0.0039	0.0025	0.069	0.0095	0.0145	0.0118	0.1057	0.0348	0.0048	0.0221	0.0269
31600	0.0006	-0.0018	0.0242	0.0025	0.0055	0.0037	0.0347	0.0096	0.0029	0.0066	0.0083
100000	-0.003	-0.0022	0.0048	0.002	0.0023	-0.0011	0.011	0.0028	-0.0009	0.0022	0.0017

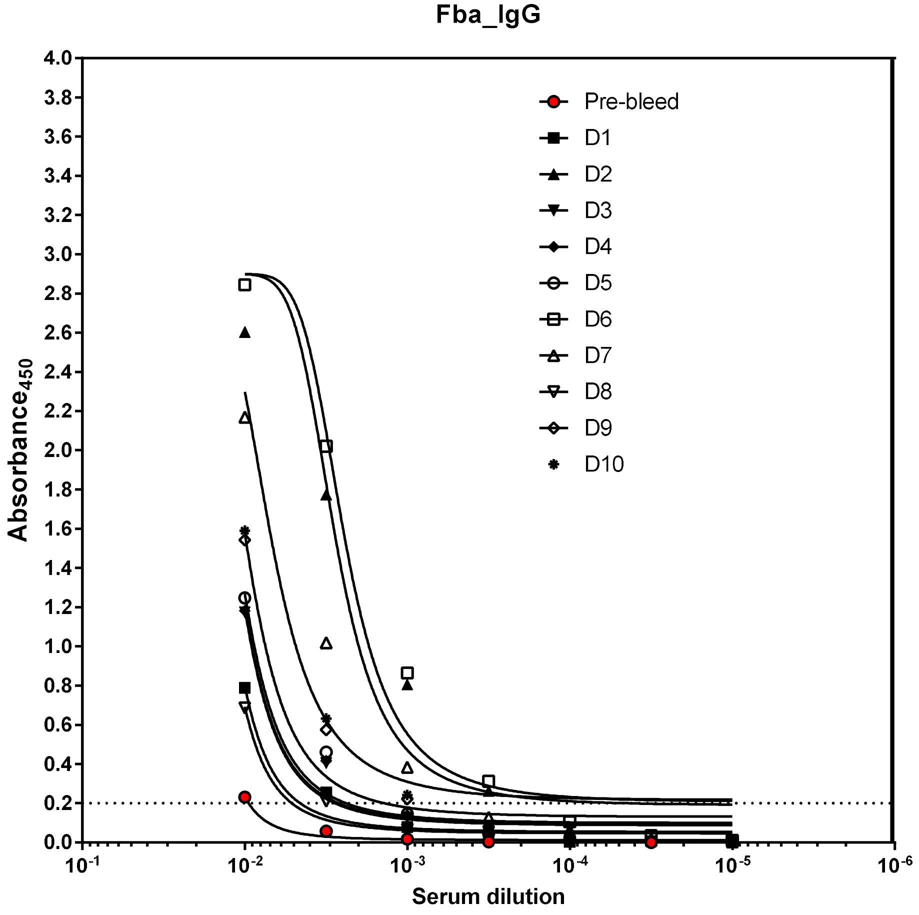

Figure S4

OD *vs* dilution: Titration curve against hexa-BSA_IgG (beta glucan hexa-saccharide)

Dilution	Pre	D1	D2	D3	D4	D5	D6	D7	D8	D9	D10
100	0.4152	0.6388	0.9831	0.94	0.88	0.8733	0.8325	1.9295	1.7253	1.532	1.5238
316	0.2924	0.2848	0.5387	0.4192	0.4446	0.3496	0.3965	0.8002	0.6792	0.5975	0.6489
1000	0.1547	0.1334	0.2169	0.178	0.2057	0.2503	0.3072	0.2879	0.2635	0.2337	0.271
3160	0.0958	0.0897	0.1139	0.0983	0.1258	0.0854	0.1065	0.1392	0.1439	0.1164	0.1305
10000	0.0691	0.0625	0.0724	0.0677	0.0734	0.0675	0.0772	0.1488	0.0844	0.0804	0.0776
31600	0.071	0.0566	0.0614	0.0574	0.059	0.0555	0.0761	0.0827	0.0625	0.0628	0.061
100000	0.0793	0.0598	0.0526	0.0555	0.0585	0.0565	0.0924	0.0662	0.0949	0.0623	0.0657

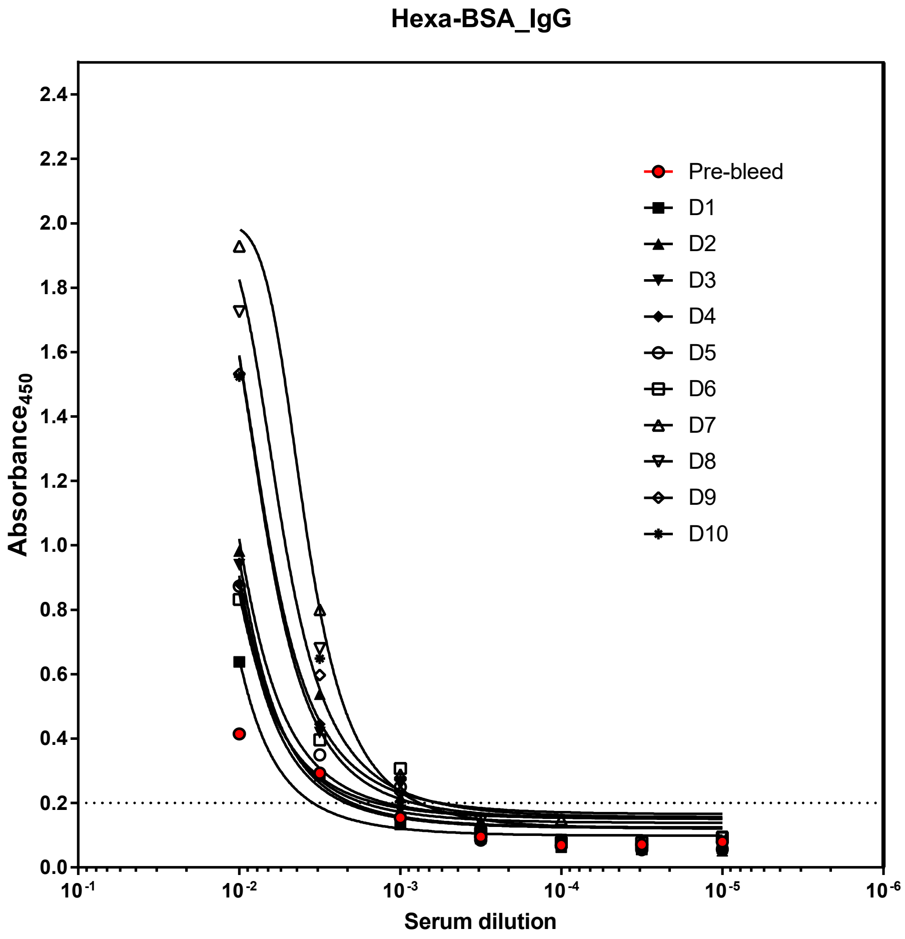

Figure S5

OD *vs* dilution: Titration curve against Hexa-Dendrimer-Ova IgG

Dilution	Pre	D1	D2	D3	D4	D5	D6	D7	D8	D9	D10
100	0.6442	1.2719	2.7298	1.9483	1.9625	2.0481	2.8776	2.6167	2.1966	2.8803	2.5721
316	0.233	0.5319	1.911	0.852	0.9118	0.9048	2.1051	1.4043	1.1179	1.4943	1.3689
1000	0.0926	0.182	0.8183	0.2896	0.3363	0.3083	0.8695	0.4844	0.3896	0.6558	0.5286
3160	0.0309	0.0639	0.291	0.1065	0.1319	0.1018	0.3089	0.1744	0.1349	0.2403	0.1957
10000	0.008	0.0187	0.0836	0.0401	0.0537	0.0404	0.108	0.0508	0.0443	0.0835	0.0539
31600	0.0045	0.0073	0.0282	0.0182	0.025	0.0129	0.0295	0.0157	0.016	0.0268	0.0198
100000	0.0398	0.0012	0.0073	0.0029	0.0054	0.0024	0.0098	0.0035	0.0041	0.0075	0.0058

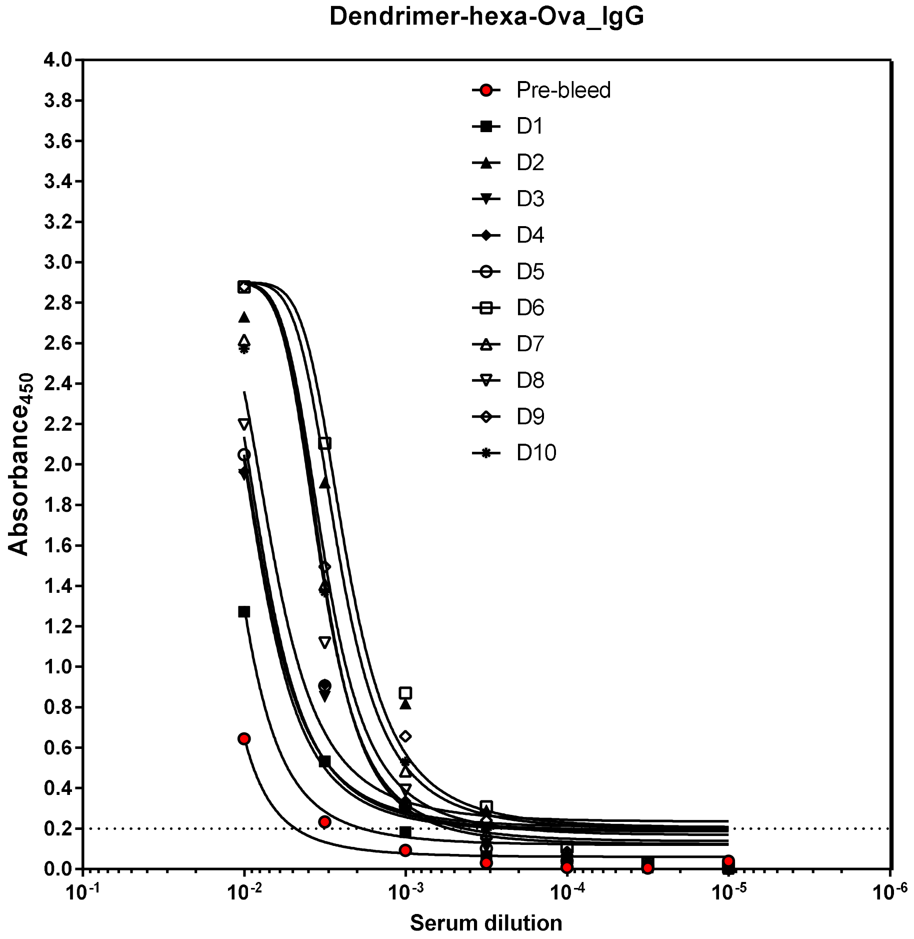

Figure S6

OD *vs* dilution: Titration curve against Native Cell wall extract gG

Dilution	Pre	D1	D2	D3	D4	D5	D6	D7	D8	D9	D10
100	0.4839	0.9134	2.752	1.3747	1.7622	1.6427	2.7849	2.2906	0.8521	1.6022	1.8488
316	0.2761	0.4495	2.2886	0.6239	0.9353	0.855	2.2184	1.4562	0.4086	0.7912	0.9431
1000	0.1001	0.1621	1.2069	0.2188	0.4082	0.3189	1.1125	0.5749	0.1515	0.3176	0.4033
3160	0.0493	0.0601	0.5198	0.086	0.1753	0.1132	0.4551	0.2117	0.0985	0.1173	0.1429
10000	0.0162	0.0295	0.1696	0.0353	0.0772	0.0381	0.1743	0.0789	0.0295	0.05	0.0591
31600	0.0122	0.003	0.0526	0.0298	0.0264	0.0189	0.0616	0.0259	0.016	0.0205	0.0185
100000	-0.004	-0.0036	0.0139	-0.0005	0.0058	-0.0011	0.015	0.004	0.0003	0.0012	0.0034

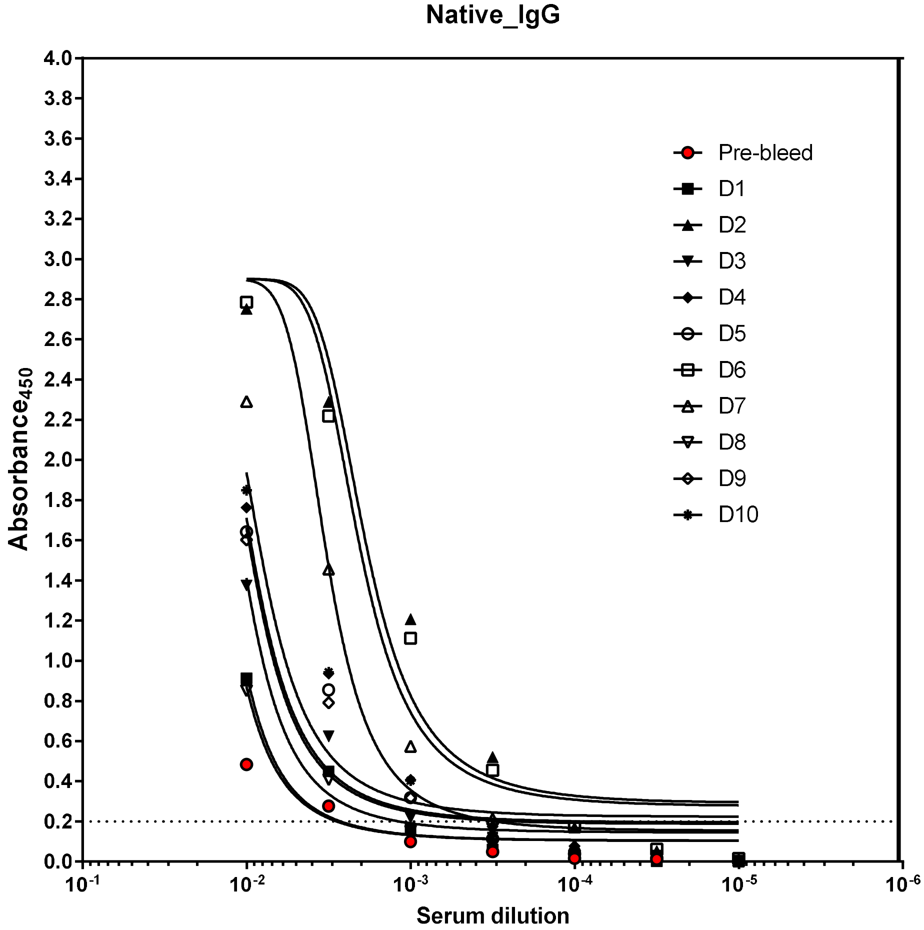

Figure S7

End point titer:
End point dilution (x0) was recorded as the serum dilution giving an absorbance 0.2 above background and end point serum titer was calculated as the reciprocal of x0. All the data were processed using Excel and Graphpad Prism software.

	Man_3	Fba	Hexa-BSA	Hexa-Den-Ova	Native
Pre bleed	71	$1 X 10^2$	$1 X 10^3$	$2 X 10^2$	$3.3 X 10^2$
D1	$2 X 10^2$	$2 X 10^2$	$5 X 10^2$	$5 X 10^2$	$3.3 X 10^2$
D2	$5 X 10^3$	$1 X 10^4$	$1 X 10^3$	$3.3 X 10^3$	$1 X 10^4$
D3	$3.3 X 10^2$	$3.5 X 10^2$	$1 X 10^3$	$3.3 X 10^3$	$1.6 X 10^3$
D4	$3.3 X 10^2$	$3.5 X 10^2$	$1 X 10^3$	$3.3 X 10^3$	$1.6 X 10^3$
D5	$3.3 X 10^2$	$3.5 X 10^2$	$1 X 10^3$	$3.3 X 110^3$	$1.6 X 10^3$
D6	$5 X 10^3$	$1 X 10^4$	$1 X 10^3$	$3.3 X 10^3$	$1 X 10^4$
D7	$3.3 X 10^2$	$3.3 X 10^3$	$1 X 10^3$	$3.3 X 10^3$	$1.6 X 10^3$
D8	$1.6X 10^2$	$2 X 10^2$	$1 X 10^3$	$3.3 X 10^3$	$3.3 X 10^2$
D9	$3.3 X 10^2$	$1 X 10^3$	$1 X 10^3$	$3.3 X 10^3$	$1.6 X 10^3$
D10	$3.3 X 10^2$	$3.5 X 10^2$	$1 X 10^3$	$3.3 X 10^3$	$1.6 X 10^3$

B: TT-Fba-Man3 immunised mice:

OD *vs* dilution: Titration curve against Man_3 IgG

Dilution	Pre	T1	T2	T3	T4	T5	T6	T7	T8	T9	T10
100	1.429	2.5599	1.5436	2.7956	1.4362	2.479	1.8579	0.9292	2.195	1.9131	2.4937
316	0.661	2.3204	0.7958	2.1735	0.6006	1.6938	0.8834	0.4379	1.1179	0.9437	1.6526
1000	0.183	1.1295	0.2805	0.8622	0.1741	0.6126	0.2811	0.1373	0.3414	0.3015	0.6495
3160	0.062	0.345	0.1031	0.2617	0.0536	0.1668	0.0812	0.0466	0.0875	0.0745	0.1786
10000	0.018	0.1208	0.1288	0.0763	0.0141	0.0414	0.0202	0.0135	0.0243	0.0187	0.0418
31600	0	0.043	0.1027	0.0246	0.0041	0.0113	0.0105	0.0049	0.0083	0.0047	0.0133
100000	-0.012	0.0221	0.0079	0.0097	0.0024	0.0071	0.0122	0.0002	0.0106	0.0016	0.0092

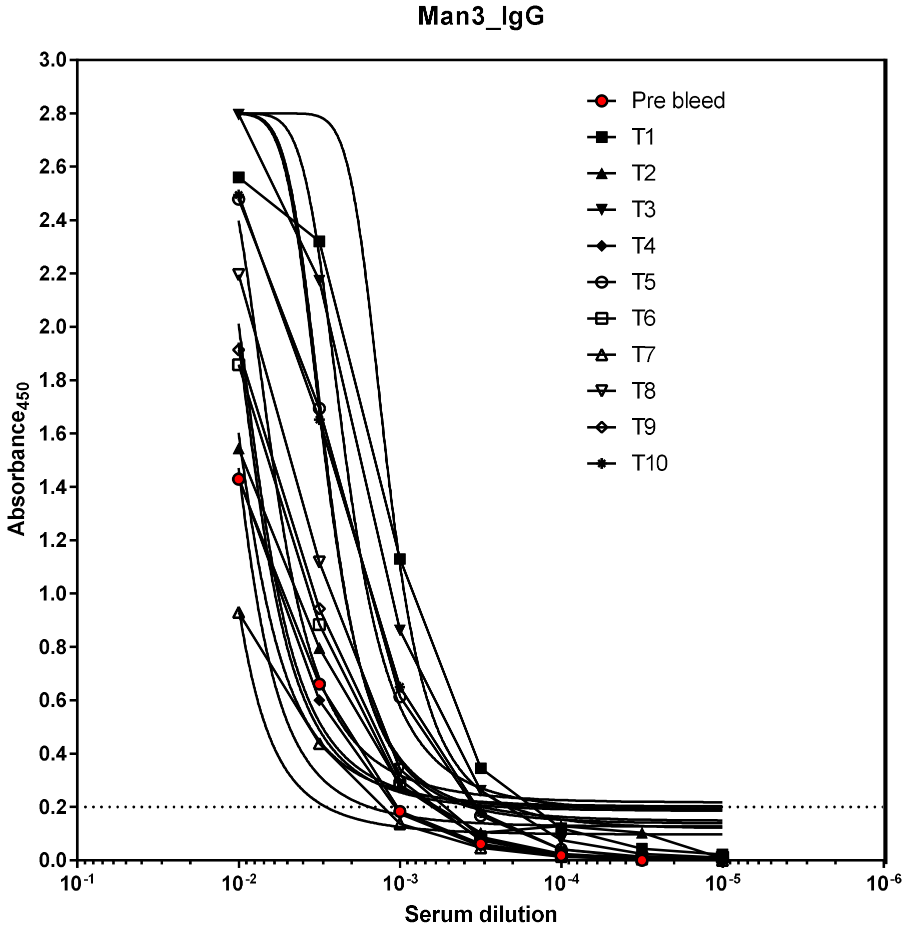

Figure S8

OD *vs* dilution: Titration curve against Fba_IgG

Note: some titration curves were extrapolated (manually) to the 0.2 line to record the end point dilution.

Dilution	Pre	T1	T2	T3	T4	T5	T6	T7	T8	T9	T10
100	0.588	2.8487	3.301	3.2749	3.2797	3.2498	3.0387	3.2737	3.237	3.2767	3.3044
316	0.279	2.108	3.3395	3.3029	3.3263	3.2664	2.4359	3.3017	3.2523	3.2719	3.3273
1000	0.163	0.9579	3.3846	3.2713	3.3103	3.2144	1.2658	3.2179	3.2106	3.1394	3.2979
3160	0.118	0.3259	3.3728	3.0063	3.0957	2.9468	0.4693	2.8592	2.8982	2.4785	3.0693
10000	0.105	0.105	3.316	2.0514	2.2429	1.9532	0.1606	1.7831	1.8024	1.1692	2.1772
31600	0.108	0.0301	2.9442	0.868	0.9864	0.8886	0.0564	0.6911	0.7248	0.42	0.9175
100000	0.109	0.041	1.7803	0.3037	0.3475	0.3	0.0527	0.2349	0.2501	0.1368	0.3226

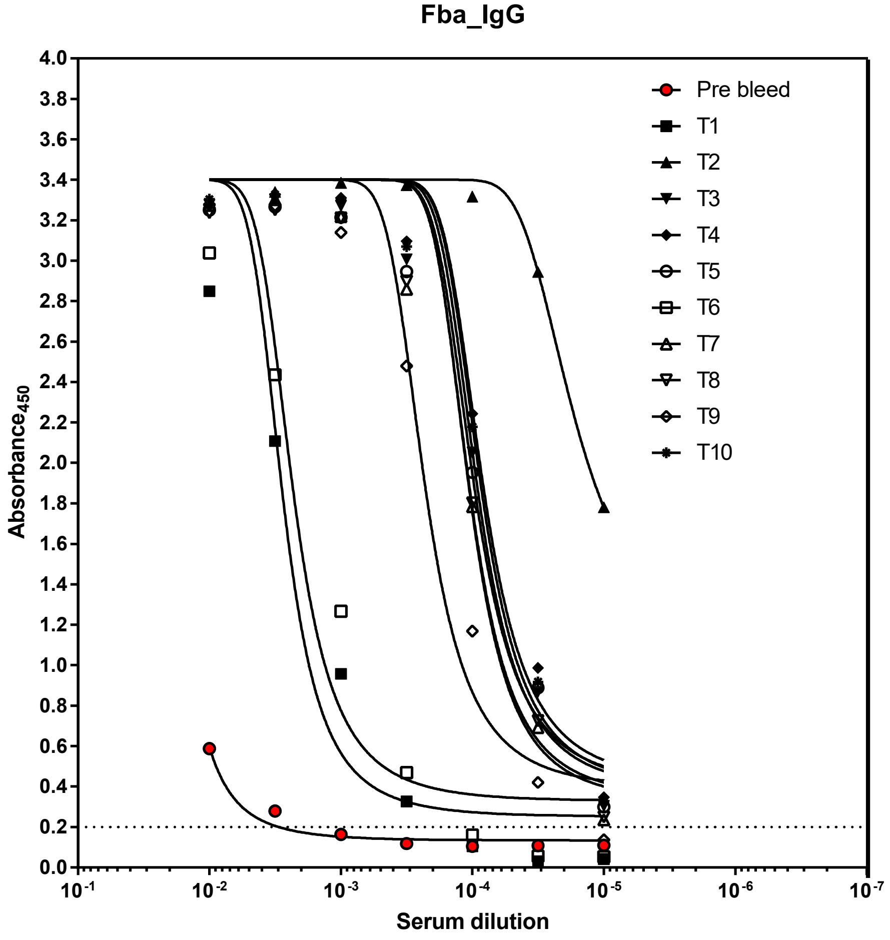

Figure S9

OD *vs* dilution: Titration curve against Native IgG

Due to the limited supply of the native antigen only five sera were screened.

Dilution	Pre	T1	T3	T5	T8	T10
100	1.798	0.3184	1.6726	2.3239	1.8571	1.7719
316	1.1188	0.1497	1.3095	1.7535	1.1619	0.9769
1000	0.4353	0.065	0.6495	0.8428	0.4909	0.3571
3160	0.1576	0.0308	0.2126	0.3175	0.1752	0.1333
10000	0.0528	0.0229	0.0793	0.112	0.0577	0.071
31600	0.0122	0.0017	0.0173	0.035	0.0145	0.0103
100000	0.0003	-0.0039	0.0003	0.0062	0	-0.0017

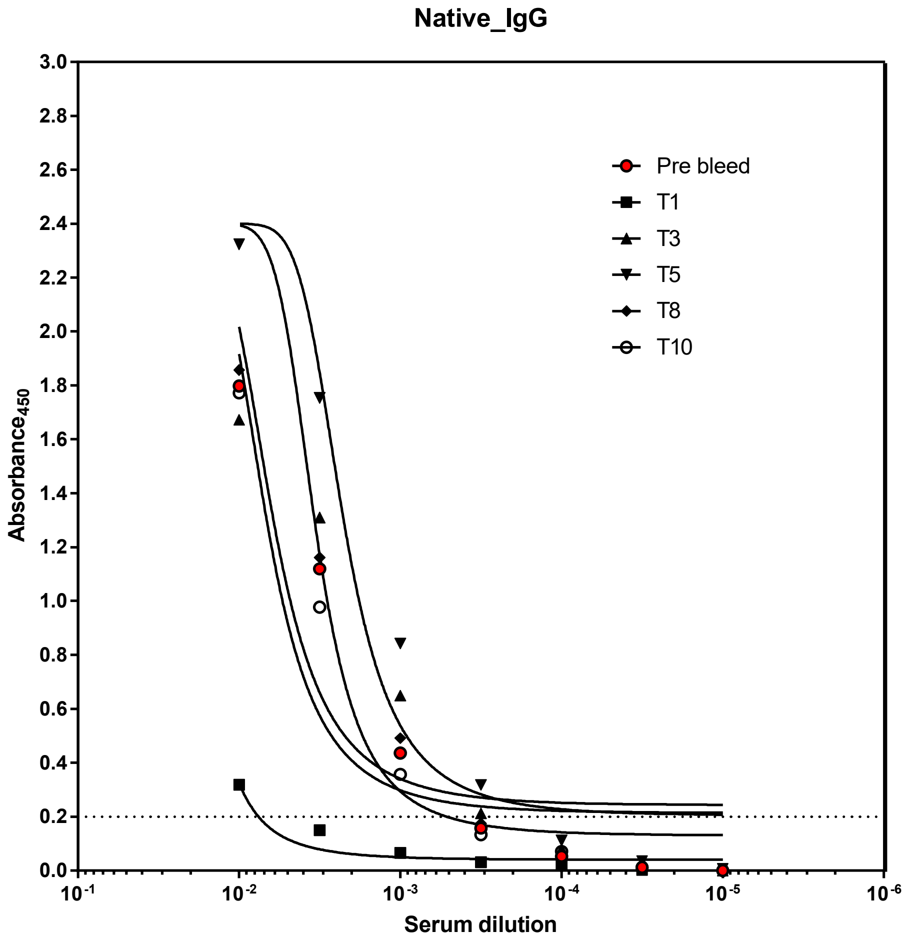

Figure S10

End point titers

	Man3	Fba	Native
Pre bleed	1×10^3	2.9×10^2	2×10^3
T1	5×10^3	5×10^3	120
T2	1×10^3	5×10^6	x
T3	5×10^3	5×10^5	3.1×10^3
T4	5×10^3	5×10^5	x
T5	5×10^3	5×10^5	5×10^3
T6	5×10^3	1×10^4	x
T7	1×10^3	5×10^5	x
T8	5×10^3	5×10^5	3.1×10^3
T9	5×10^3	5×10^5	x
T10	5×10^3	5×10^5	2×10^3

T test: In prism, we plotted the end point titers for both dendrimer conju and TT conj immunised sera. Then Mann-Whitney t-test (non parametric distribution) was performed.

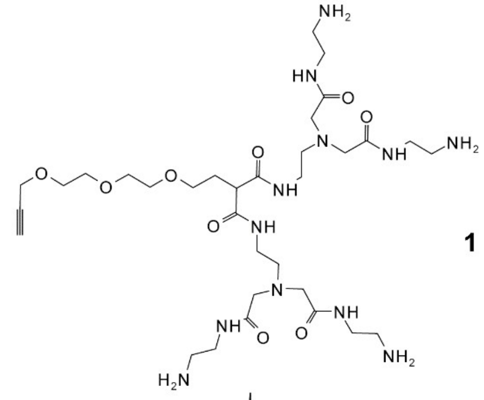

1

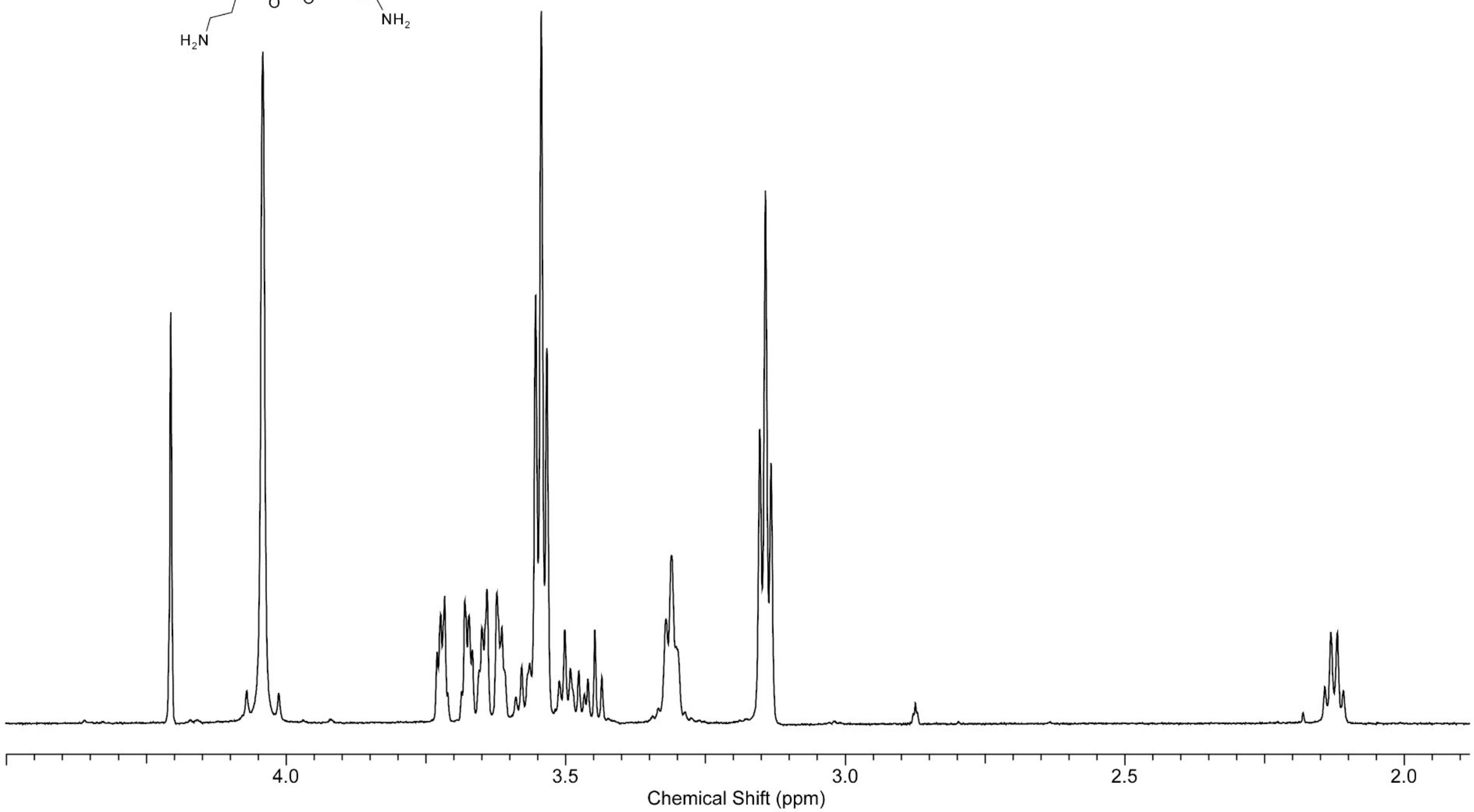

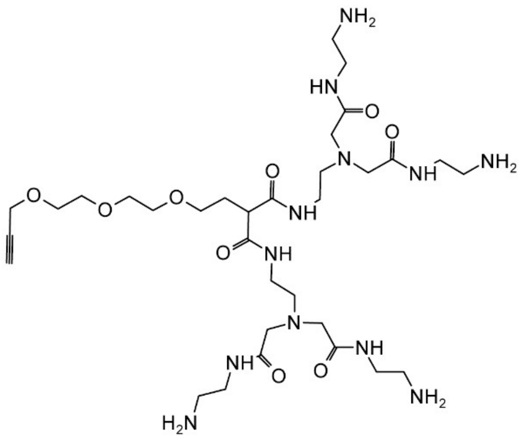

NH2
HN
O
O
N
NH
NH2
O
O
O
O
NH
O
NH
N
NH
O
O
NH
NH2
H2N

1

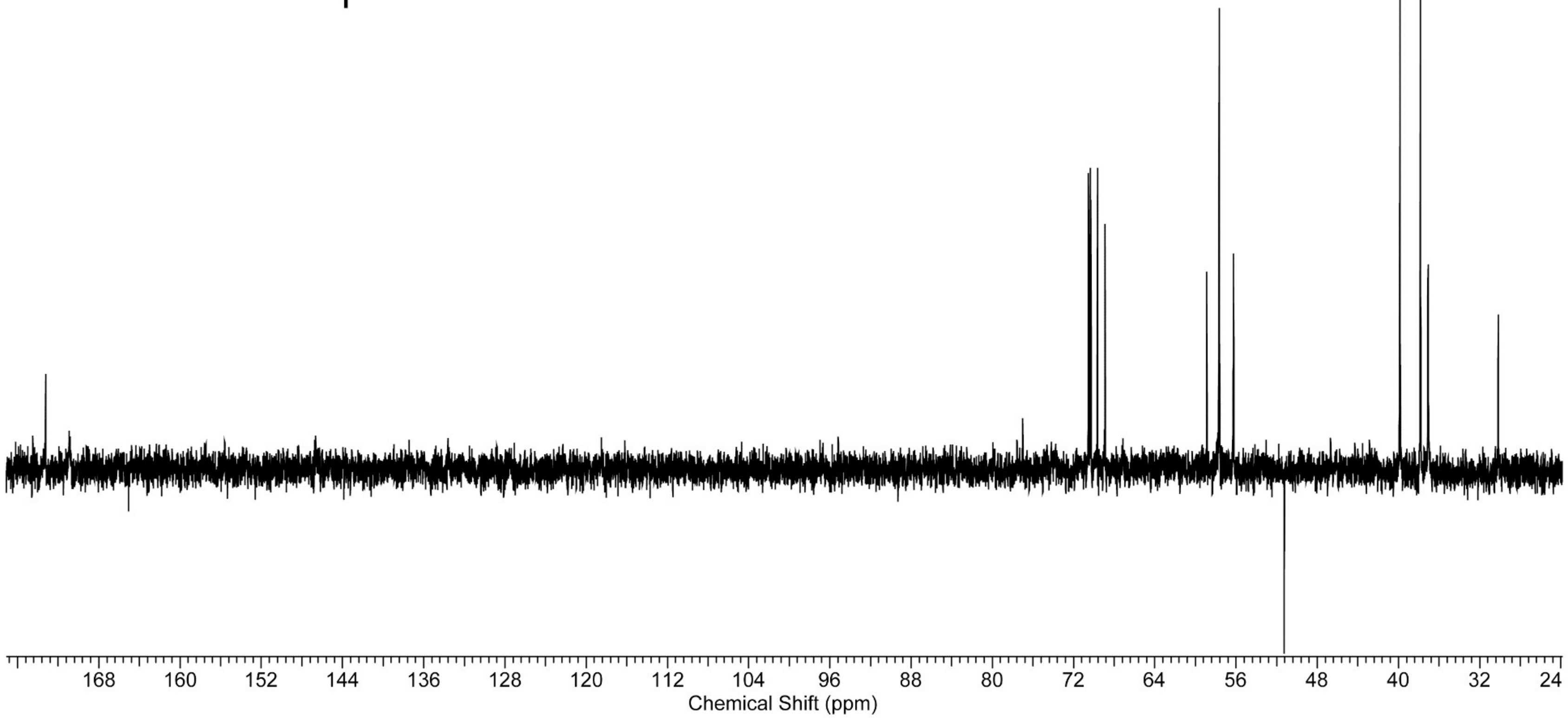

168
160
152
144
136
128
120
112
104
96
88
80
72
64
56
48
40
32
24
Chemical Shift (ppm)

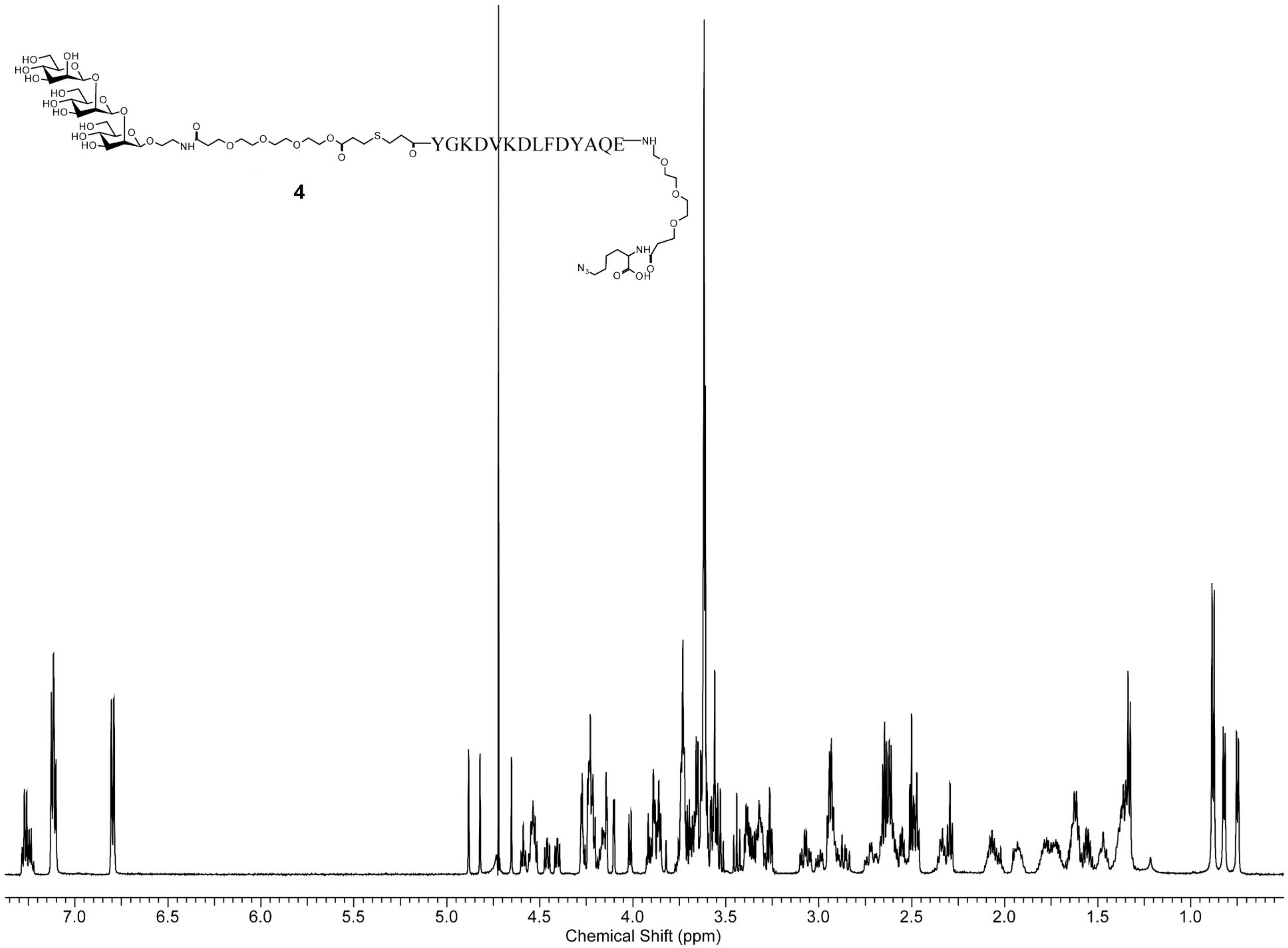
YGKDVKDLFDYAQE
4
7.0
6.5
6.0
5.5
5.0
4.5
4.0
3.5
3.0
2.5
2.0
1.5
1.0
Chemical Shift (ppm)

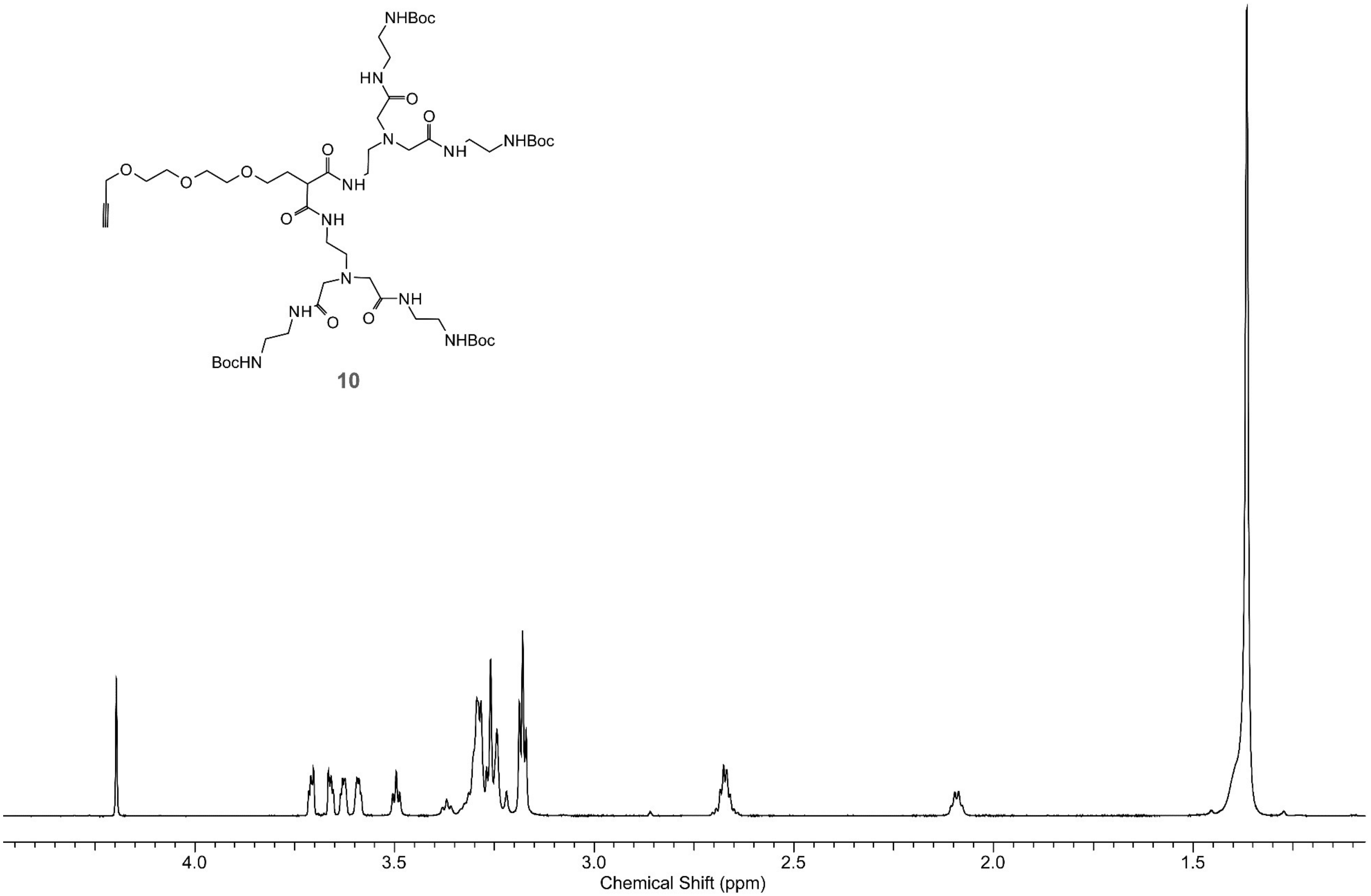
NHBoc
HN
O
O
N
NH
NHBoc
O
O
O
O
NH
O
NH
N
NH
O
O
NH
BocHN
NHBoc
10
4.0
3.5
3.0
2.5
2.0
1.5
Chemical Shift (ppm)

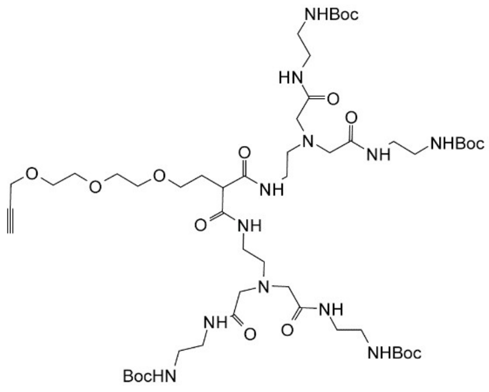
NHBoc
HN
O
O
NH
NHBoc
O
O
O
O
N
NH
O
NH
N
NH
O
O
NH
NHBoc
BocHN

10

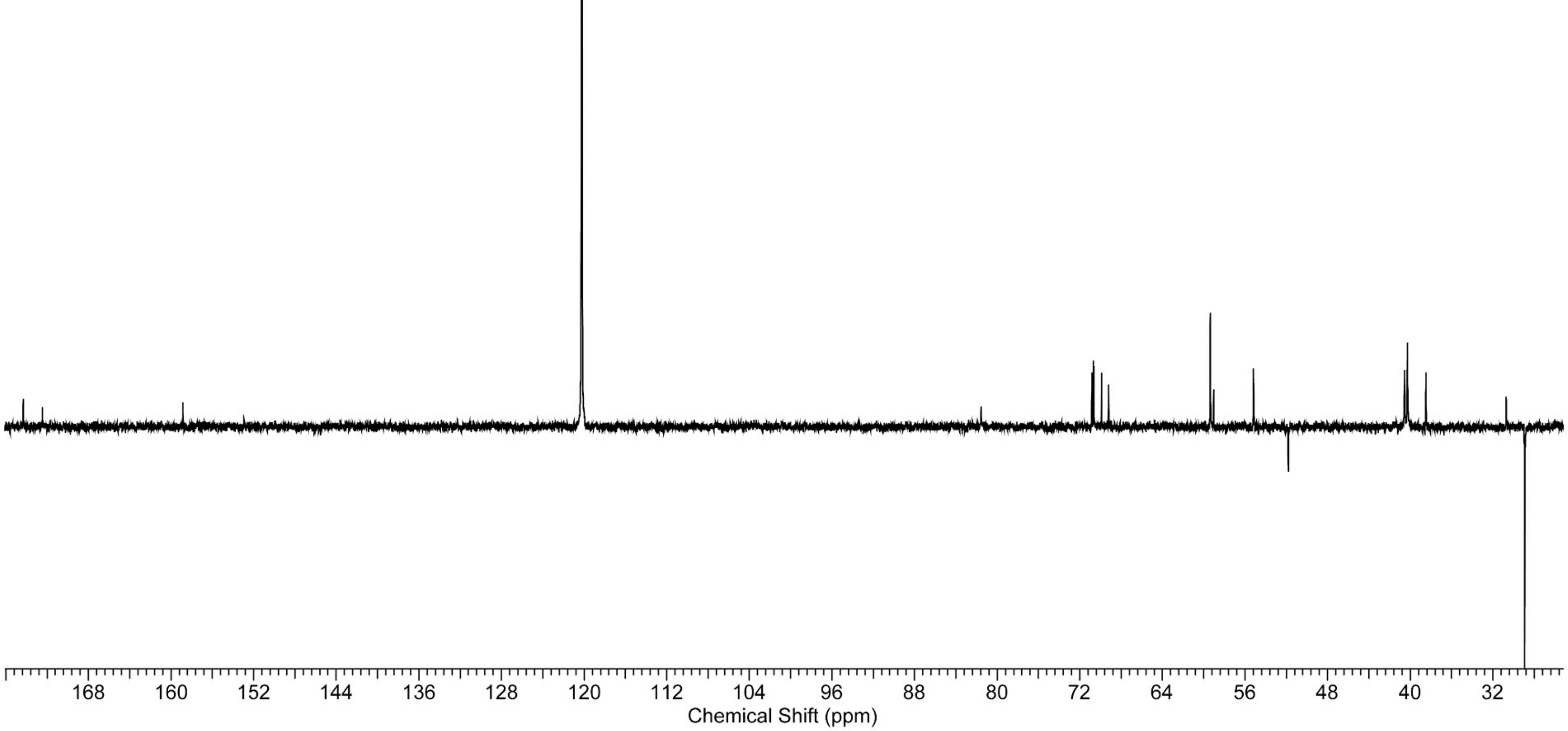
168
160
152
144
136
128
120
112
104
96
88
80
72
64
56
48
40
32
Chemical Shift (ppm)

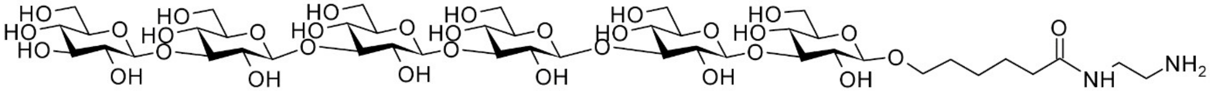

19

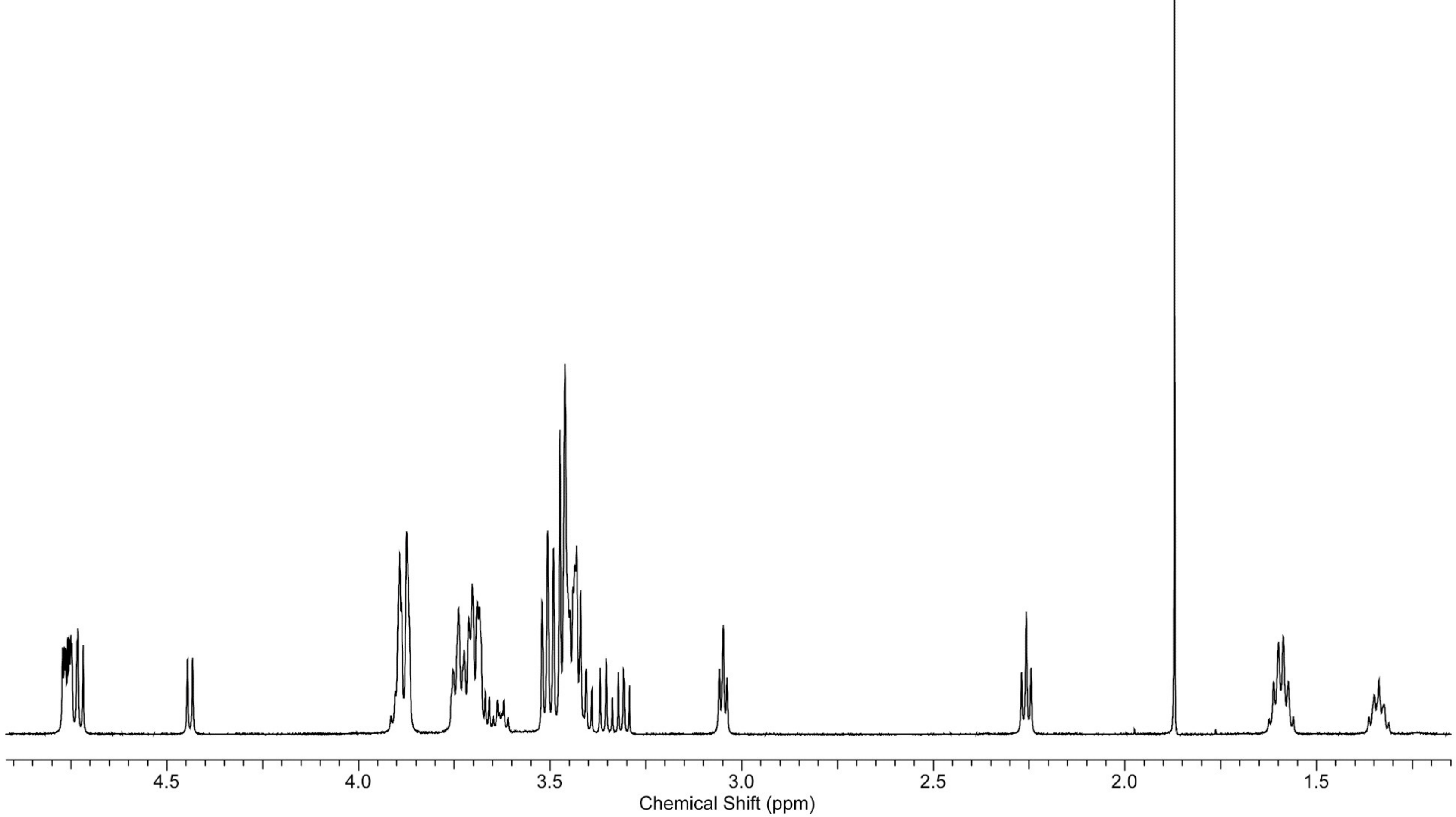

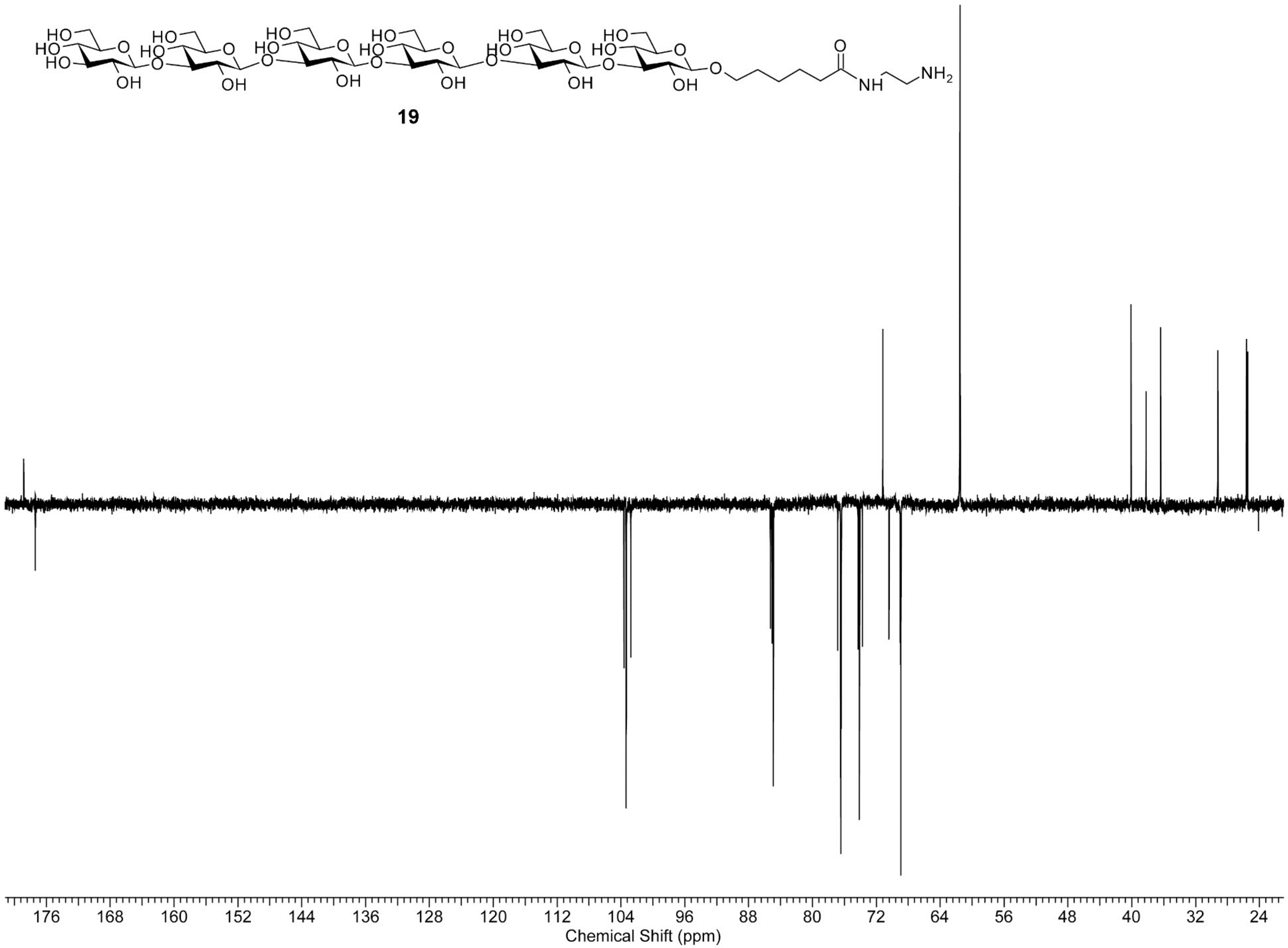
19
NH
NH2
176 168 160 152 144 136 128 120 112 104 96 88 80 72 64 56 48 40 32 24
Chemical Shift (ppm)

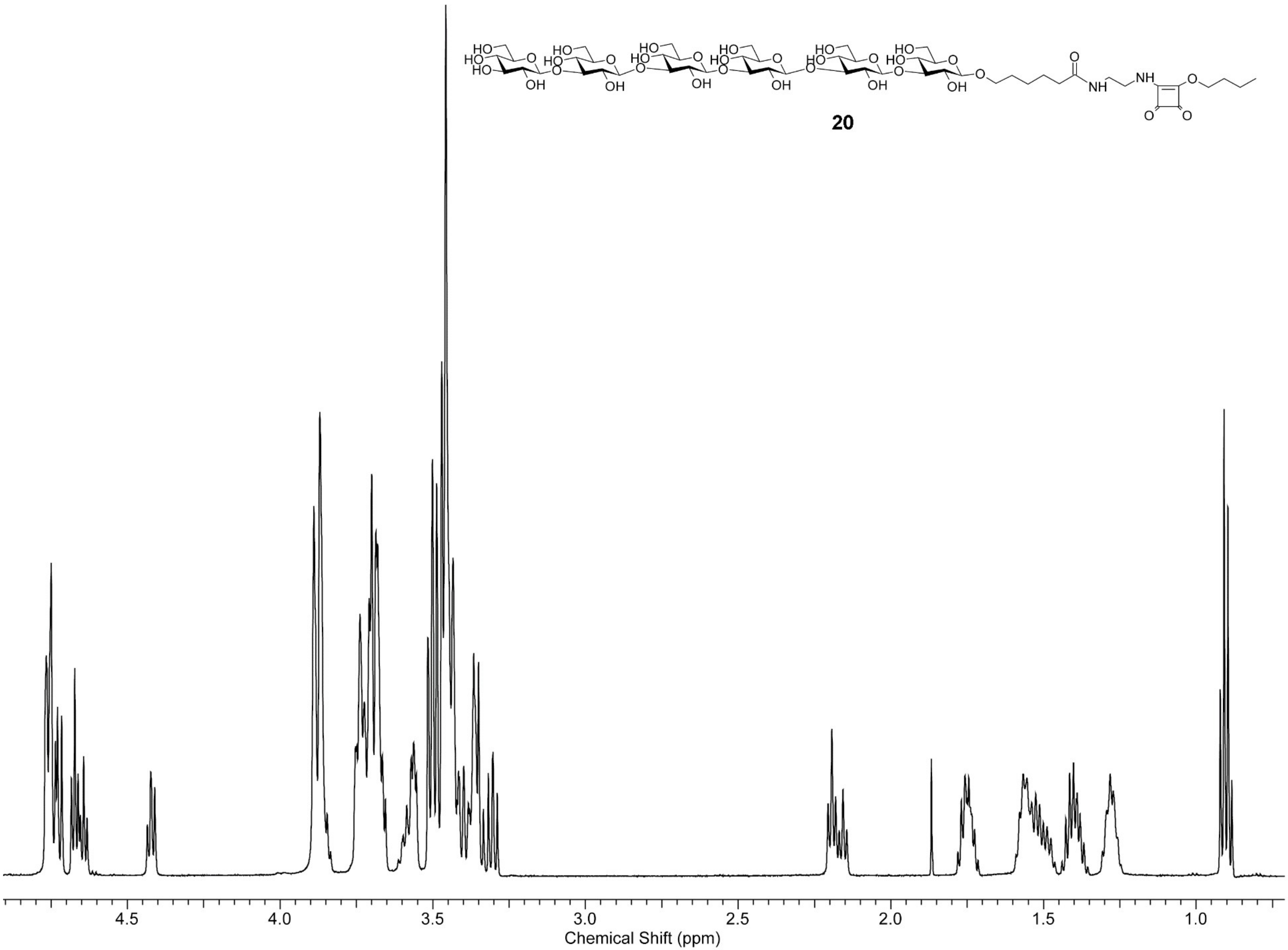

20
4.5
4.0
3.5
3.0
2.5
2.0
1.5
1.0
Chemical Shift (ppm)

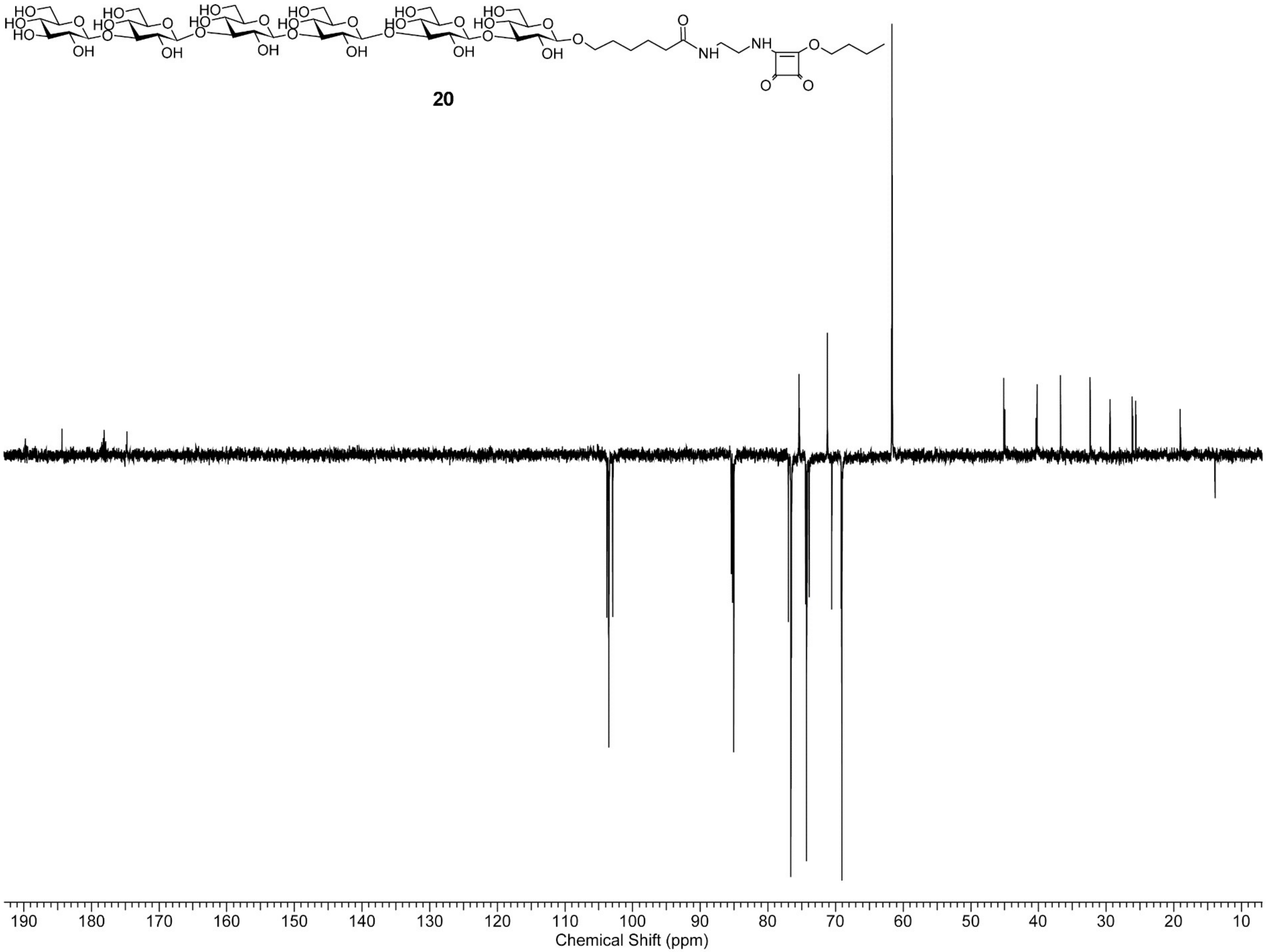
20
Chemical Shift (ppm)

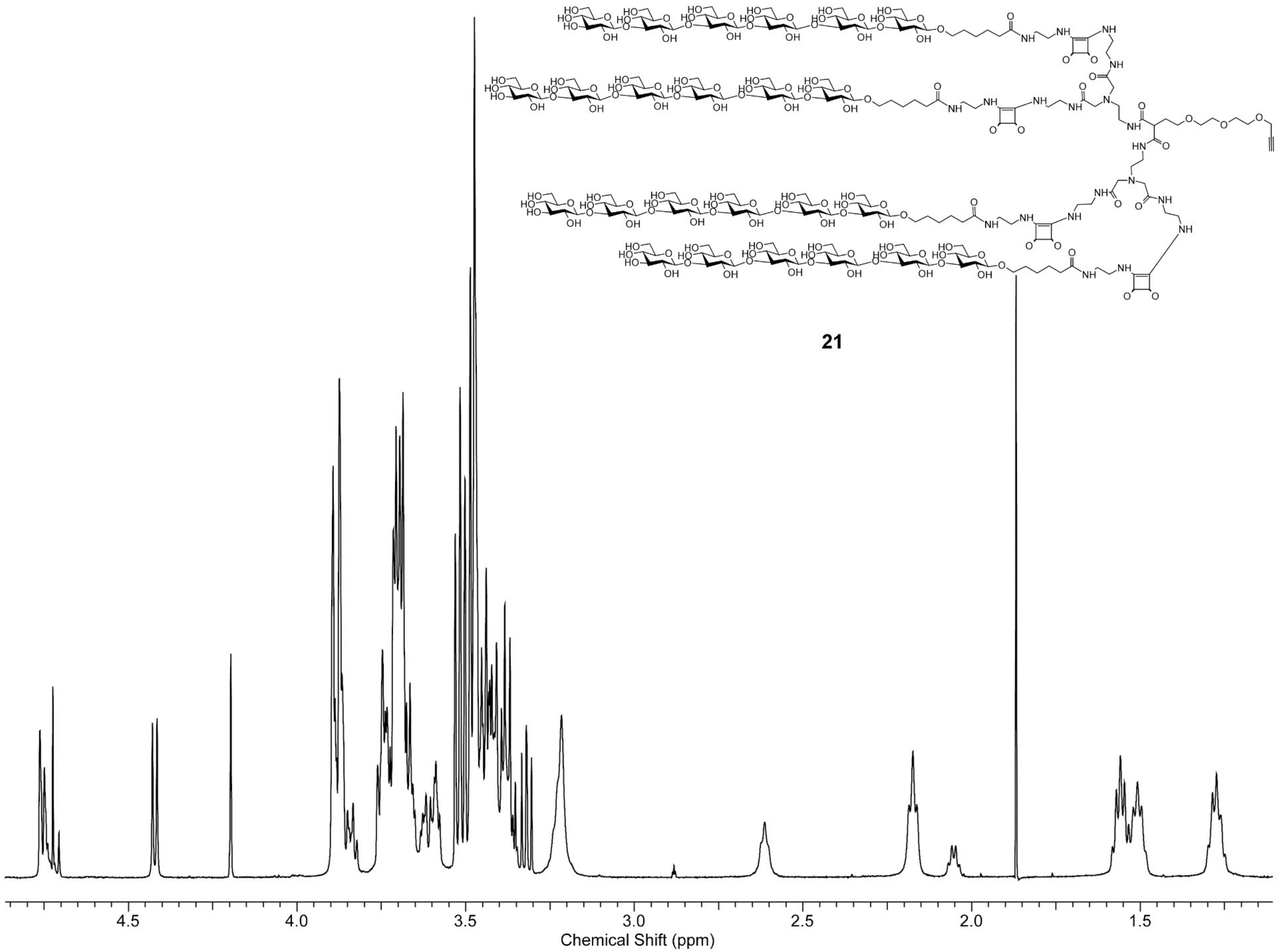
21
4.5
4.0
3.5
3.0
2.5
2.0
1.5
Chemical Shift (ppm)
